# ProFAT: a web-based tool for the functional annotation of protein sequences

**DOI:** 10.1186/1471-2105-7-466

**Published:** 2006-10-23

**Authors:** Charles Richard Bradshaw, Vineeth Surendranath, Bianca Habermann

**Affiliations:** 1 Max Planck Institute of Molecular Cell Biology and Genetics, Pfotenhauerstrasse 108, 01307 Dresden, Germany; 2 Scionics Computer Innovation, GmbH, Tatzberg 47–51, 01307 Dresden, Germany

## Abstract

**Background:**

The functional annotation of proteins relies on published information concerning their close and remote homologues in sequence databases. Evidence for remote sequence similarity can be further strengthened by a similar biological background of the query sequence and identified database sequences. However, few tools exist so far, that provide a means to include functional information in sequence database searches.

**Results:**

We present ProFAT, a web-based tool for the functional annotation of protein sequences based on remote sequence similarity. ProFAT combines sensitive sequence database search methods and a fold recognition algorithm with a simple text-mining approach. ProFAT extracts identified hits based on their biological background by keyword-mining of annotations, features and most importantly, literature associated with a sequence entry. A user-provided keyword list enables the user to specifically search for weak, but biologically relevant homologues of an input query. The ProFAT server has been evaluated using the complete set of proteins from three different domain families, including their weak relatives and could correctly identify between 90% and 100% of all domain family members studied in this context. ProFAT has furthermore been applied to a variety of proteins from different cellular contexts and we provide evidence on how ProFAT can help in functional prediction of proteins based on remotely conserved proteins.

**Conclusion:**

By employing sensitive database search programs as well as exploiting the functional information associated with database sequences, ProFAT can detect remote, but biologically relevant relationships between proteins and will assist researchers in the prediction of protein function based on remote homologies.

## Background

Functional prediction of experimentally uncharacterized proteins is an important research area in bioinformatics. On the one hand, the functional prediction of a protein can help in advancing biological science by generating testable hypothesis for experimental research. On the other hand, it improves the annotation of sequenced genomes by assigning functional information to predicted genes. Functional prediction relies mostly on the similarity between sequences and standard sequence similarity search tools have been successfully applied in protein functional annotation, provided that the similarity between related proteins is significant enough for sequence-based detection. If, however, the similarity between related protein sequences is low, profile-based database search methods like PSI-BLAST or HMMer, as well as fold recognition tools have proven successful in detecting remote homologies and therefore can assist in predicting the function of uncharacterized proteins [1,2]).

Experimentally characterized proteins have extensive functional information associated with their sequence records. This functional information includes published literature about a protein or gene, functional classifications as for instance provided by the Gene Ontology (GO-) annotations, conserved domains that potentially link a protein with a molecular function, and sometimes even a short summary about the proteins' function. Given detectable sequence similarity between a functionally characterized and an uncharacterized protein, this information can be used to predict the putative function of the unknown protein. Given the complexity of the output of sequence-, as well as structure-based search techniques, the exploitation of this functional knowledge is often tedious and involves extensive manual mining for the biological context of identified database sequences.

With the advancement of experimental techniques in molecular biology, for example biochemical screens (co-immuno-precipitation experiments) and functional screens, so far uncharacterized proteins can often be put into the context of a cellular process while their molecular function may not become obvious by standard sequence similarity search tools. The combination of sensitive similarity search techniques with functional annotation of identified hits has already been successfully applied for the functional prediction of proteins, where the biological context of the protein of interest could be assigned based on the existing experimental information of remote homologues [3,4]. Similarity search tools are however generally restricted to the usage of sequence or structural information of proteins and tend to neglect functional information associated with the sequences under analysis, which could be utilized to aid bioinformatics analysis. By performing text-mining on the functional annotation associated with a sequence record, this information can be combined with traditional database search algorithms to filter identified hits based on their biological relevance. However, few efforts so far exist that incorporate functional information in similarity search methods. The program SAWTED (Structure Assignment With Text Description) uses text descriptions from the SWISS-PROT database to circumvent the problem of post-filtering of PSI-BLAST results [5]. OntoBLAST, as another example, takes advantage of the Gene Ontology based annotation of protein sequences to divide BLAST-outputs according to Gene Ontology (GO-) terminology [6].

With ProFAT, we introduce a tool that combines a remote sequence similarity search tool (PSI-BLAST, [7]) with fold recognition (Threader 3.5 [8]) and a text-mining algorithm to extract identified hits of both programs based on their biological function. In addition to Gene Ontology and GenBank feature annotations provided by the NCBI, ProFAT mines the literature associated with a sequence database entry for post-filtering of identified hits based on their biological context and is therefore the first tool that takes advantage of the wealth of published information associated with sequence database entries. The user can furthermore selectively extract hits identified by PSI-BLAST and Threader 3.5 based on a specific biological context by providing a user-specific keyword list, which makes ProFAT customizable to any biological setting. In addition to post-filtering of PSI-BLAST and Threader results for a user-specified biological context, ProFAT also provides a fully annotated output containing all identified hits, which eases the often time-consuming manual mining of literature and sequence record annotations.

## Implementation

**Figure 1:**
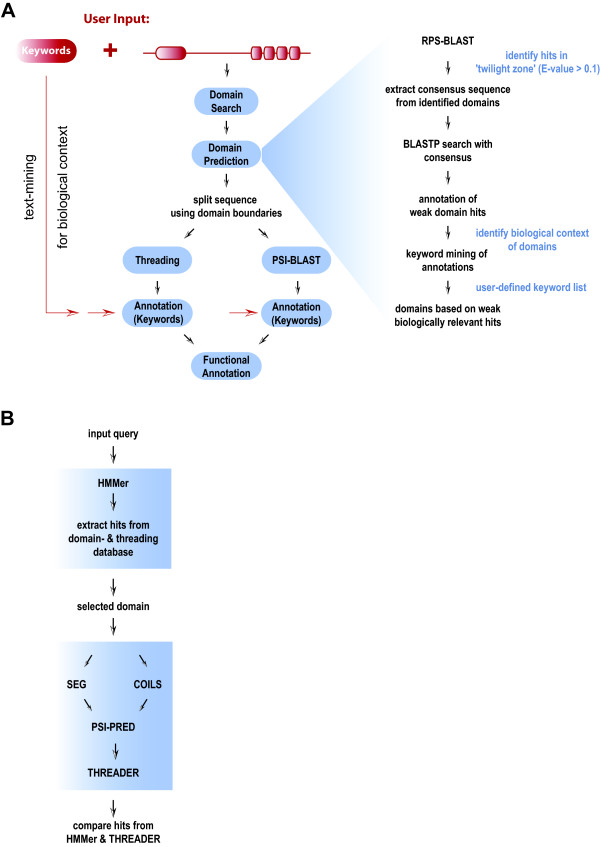
**Workflow of a ProFAT Analysis**. **(A) **A protein sequence and a keyword list are required inputs for a ProFAT analysis. The first step carried out by ProFAT is a domain search (RPS-BLAST) against the CDD-database from the NCBI. If no conserved domain is detected with RPS-BLAST, the user can proceed to domain prediction (A, right figure), which combines a RPS-BLAST search with relaxed parameters with a BLAST-search and subsequent text-mining for the biological relevance of identified hits. Alternatively, the user can choose to split the sequence into fragments between 150 and 300 amino acids for further processing. Selected conserved domains and/or regions of the input query can then be submitted to the *Annotation Engine *and/or *Threading Engine*. The *Annotation Engine *combines a PSI-BLAST search with text-mining of Gene Ontology annotation, features and PubMed abstracts associated with identified hits, thereby extracting hits involved in the process/function described by the user's keyword list. The *Threading Engine *combines a Threader 3.5 run with text-mining of associated PDB-keywords, features, compound information and PubMed abstracts of identified structures for post-filtering using keywords from the user-provided keyword list. **(B) ***HMMerThread *pipeline. *HMMerThread *combines a HMMer-search against the PFAM-database of conserved domains with a Threader run. The input query is first sent to an HMMer-search, whereby only domains with an associated 3D-structure are chosen for further processing. Selected domains are then sent to Threader 3.5, with prior secondary structure prediction (PSI-PRED), coiled-coil prediction (COILS2) and low-complexity filtering (SEG), which are all performed on the entire input sequence to achieve higher accuracy. *HMMerThread *therefore can give a highly accurate prediction of conserved domains.

ProFAT combines sensitive sequence similarity search tools with text-mining for post-filtering of identified hits according to a biological process defined in the user-provided keyword list. Individual domains of a query are submitted to the search engine, rather than the full-length sequence. To achieve this, ProFAT uses standard domain searches for detection of conserved domains, whose borders are then used to split the sequence into individual regions. The resulting regions can then be selected by the user and can be submitted in parallel to a profile-based sequence similarity search tool, as well as a fold recognition tool. Associated information concerning identified hits from both pipelines is text-mined to determine the occurrence of keywords of the user-provided keyword list. Keyword-positive hits have been experimentally associated with the biological process described in the keyword list and are then selectively shown to the user. The workflow of the ProFAT server is shown in Figure 1 and is discussed in detail in Results & Discussion. The ProFAT web-server requires user intervention at each step. The user is therefore able to select domains/regions for further processing with ProFAT's *Annotation *and *Threading Engine*, as well as the *HMMerThread *pipeline, which is discussed below.

Domain searches are carried out using RPS-BLAST [9] against the CDD-database (NCBI). BLAST and PSI-BLAST [7] searches against the non-redundant protein database (NCBI) are performed using the stand-alone tools provided by the NCBI (version 2.4.17). Hidden Markov Model based domain searches (HMMer, version 2.3.2, [10]) are performed against the PFAM conserved domain database. Fold recognition is done using Threader 3.5 [8]. Secondary structure prediction prior to threading runs are performed using the program PSI-PRED [11], coiled-coil regions are detected using the program COILS2 [12], low-complexity regions are filtered using the program SEG [13]; all three programs are executed on the entire sequence with subsequent processing according to conserved domain and region boundaries. Text mining is performed using a Perl implementation for stemming from Porter [14]. For the Gene Ontology (GO-) annotation of hits, a GO-tree is constructed by aligning GO-terms of identified hits to the ontology tree provided by the GO-consortium [15].

Bulk searches of the *Annotation Engine *for the HNF-1α, PABP and PLAT domain families were run using no stemming. Only those family members that identified at least 10 hits with a keyword from the respective keyword lists were scored as positive. *HMMerThread *bulk searches were run using a threading extension of various sizes (0, 3, 5, 8, 10) for HNF-1α, 5 and 8 for PABP and 0 for PLAT family members and a threading hit depth of 20.

Multiple sequence alignments were done with ClustalX [16] and were manually refined. Structural comparisons were done using the DALI-server [17]. For testing purposes of ProFAT, we verified all the examples of weak domain hits given in this manuscript by independent PSI-BLAST searches.

## Results and discussion

### The ProFAT server

The input of a ProFAT analysis is a protein sequence and a keyword list that describes the cellular process or putative function relevant for the protein under analysis.

### ProFAT workflow

The workflow of ProFAT can be divided into 3 parts (Figure 1): a *domain search *or *domain prediction*, whereby identified conserved domains are used to split the input query for further processing with 2) the *Annotation Engine *and 3) the *Threading Engine*.

#### Domain search and prediction

**Figure 2:**
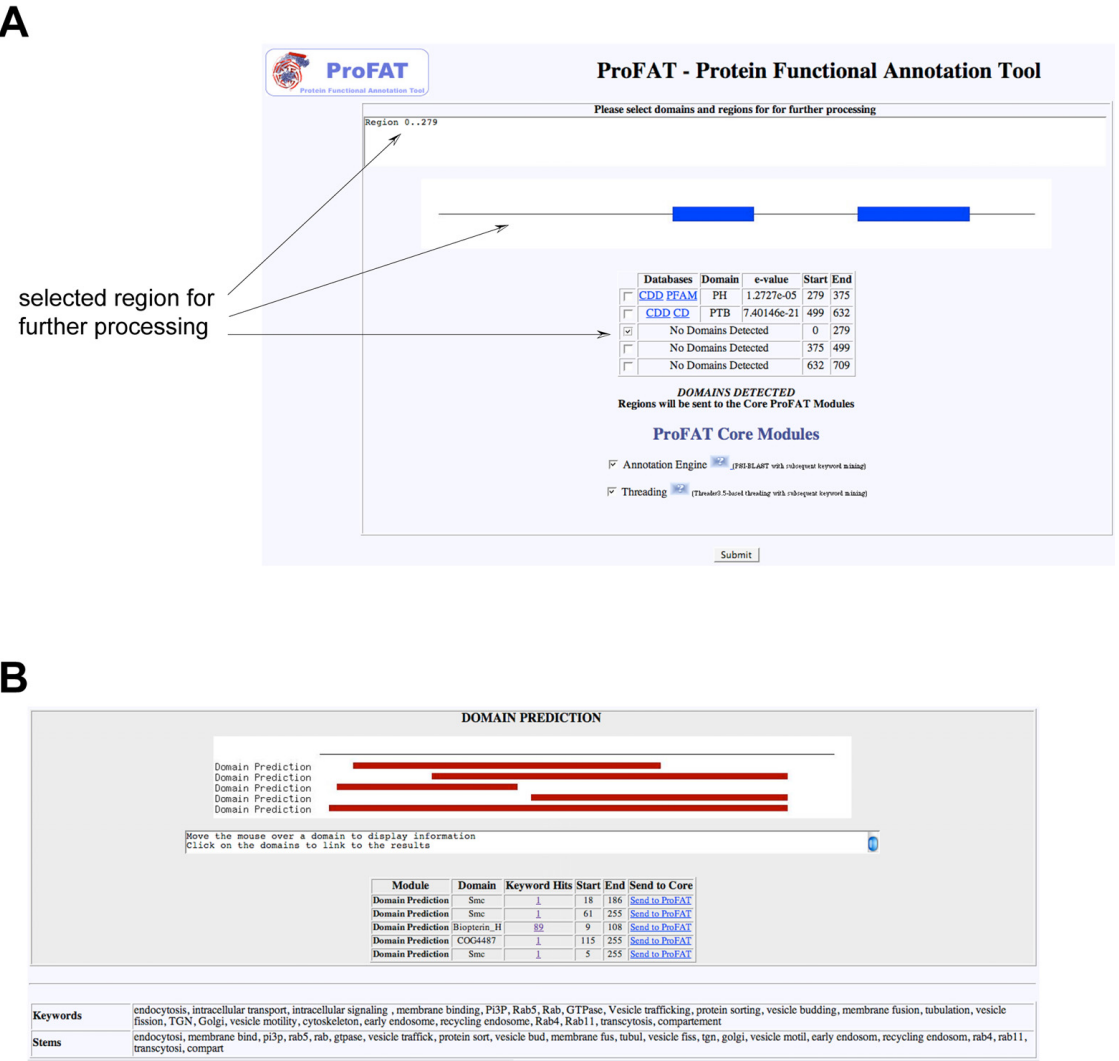
**Domain search and domain prediction using ProFAT**. **(A) **Results for Dip13α/APPL1 [GenBank:NP_036228] from a ProFAT domain search. RPS-BLAST identified a PH-domain and a PTB-domain in the input query, the N-terminal region does not contain any conserved domains with the chosen E-value cutoff (E <= 1E-04). The upper window gives the user a description of the domain as found in CDD by mousing over the domain box. The image represents the sequence with identified conserved domains. The table at the bottom lists the identified domains with their amino acid boundaries in the input sequence. By either activating the checkboxes or by clicking on the region and/or conserved domain on the image, the user can select conserved domains/regions for further processing by the *Annotation Engine *and/or *Threading Engine *(selectable by a check-box and activated by pressing the 'Submit' box). In this case, the N-terminal region from amino acid 1 to 280 was selected for further processing. **(B) **If no domain was identified, the user can perform *Domain Prediction*. In this case, a RPS-BLAST search with an E-value cutoff of 100 is used to identify weak domain hits. The consensus sequences of these domains are, in turn, submitted to a regular BLAST-search with subsequent text-mining for keywords occurring in the user-provided keyword list. In the case of the Dip13α/APPL1 N-terminal domain, RPS-BLAST finds SMC-domains, Biopterin_H, as well as a COG-domain. The identified domains can be submitted to the *Annotation Engine *and *Threading Engine *for a more detailed analysis (link 'Send to ProFAT').

Initial domain searches (using RPS-BLAST against the CD-database (NCBI)) are carried out using a restrictive E-value cutoff (E <= 1E-04) by default. Identified conserved domains can be selected in the results page for further processing. Figure 2A shows the results of a domain search for the protein Dip13α/APPL1 [GenBank:NP_036228]. In this case, ProFAT identified a central PH-domain and a C-terminal PTB domain in the input query. If the domain search fails to identify conserved domains, the user can perform a domain prediction. In this case, RPS-BLAST is run using relaxed settings (E <= 100). Identified weak domain hits are subsequently submitted to a BLAST-search, whereby the resulting hits are mined for their biological relevance using the user-provided keyword list. Figure 2B shows the results of the domain prediction for the N-terminal 280 amino acids of Dip13α/APPL1. If both approaches fail to detect conserved domains, the input sequence can be split into fragments of sizes between 150 and 300 amino acids.

At this stage, selected domains can be in parallel submitted to the *Annotation Engine*, the *Threading Engine*, as well as to an *HMMerThread *run for a keyword-independent domain prediction.

#### Annotation engine

**Figure 3:**
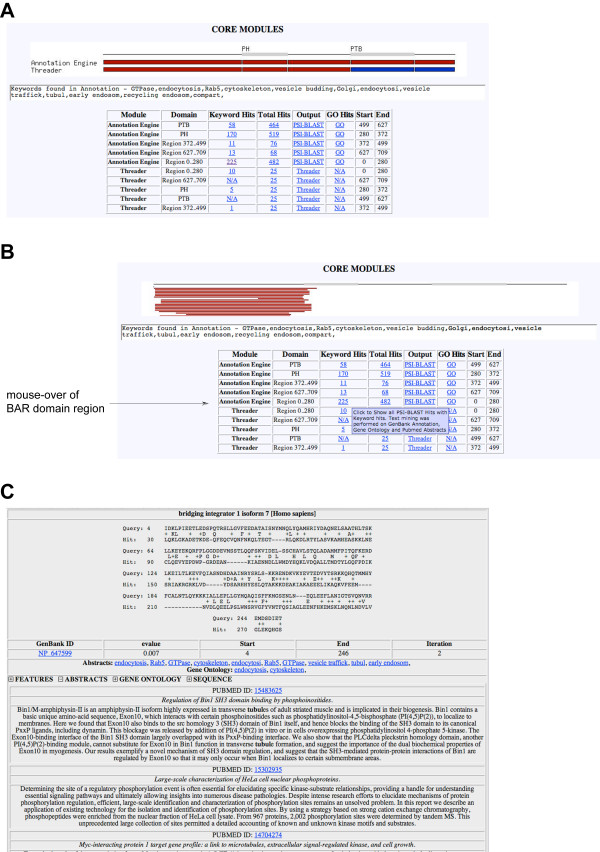
**Results from the Core Modules of ProFAT**. **(A) **Graphical and tabular representation of results from the *Annotation Engine *and *Threading Engine *(Dip13α/APPL1 [GenBank:NP_036228] was used as a query). Red bars in the image represent identified database sequences that contain one or more keywords from the user-provided list in their annotation, blue bars represent sequences where no keywords were detected. The upper bars show results from the *Annotation Engine*, the lower bars those from the *Threading Engine*. The table below the image gives the user the number of hits with and without keywords, links to the raw results, tabular information on the frequency of observed GO-terms, as well as the starting and ending residue of the region and conserved domains in the input query. The numbers in the column 'Keyword Hits' link to the annotated alignments of keyword-positive database entries. Moving the mouse over the respective number changes the format of the graph to the image seen in (B), whereby alignments are represented by narrow lines. The number in 'Total Hits' links to the complete PSI-BLAST output, whereby each alignment is annotated with the associated information of the database hit. **(B) **Graphical output of the region 1 – 280 of the input query from the *Annotation Engine*. **(C) **Representative alignment of one of the identified hits that shows biological relevance next to sequence similarity. Each sequence that has been identified by PSI-BLAST is annotated with associated GenBank features, PubMed abstracts and Gene Ontology terms, as well as its sequence. Associated information can be individually viewed by clicking on the '+' sign next to the respective information.

The *Annotation Engine *sends the selected conserved domains and/or regions of the input query to a PSI-BLAST search against the non-redundant database. Associated information including GO-annotation, GenBank features, as well as associated publication abstracts of identified hits are subsequently text-mined for the occurrence of user-provided keywords. Figure 3 shows a typical output from the *Annotation Engine*, where full-length Dip13α/APPL1 was used as the query sequence with a keyword list tailored for '*Endocytosis*' [see Additional file 1]. Results from the *Annotation Engine *for each domain are represented by red bars – when keywords of the user-provided list are found in information associated with identified database entries – and by blue bars – when none of the keywords have been detected (top bars in Figure 3A). Results from the *Threading Engine *are represented in a similar fashion (bottom bars in Figure 3A). The output of both core modules is highly interactive. Mouse-over of the BAR-domain region of Dip13α/APPL1 (N-terminal red bar or region 1–280 in the associated table of the *Annotation Engine*) results in a graphical representation of the individual alignments (Figure 3B). By clicking on the region bar or the number in the column 'Keyword Hits' in the associated table the user gets access to the post-processed alignments from the PSI-BLAST search (Figure 3C). The user can individually access the information associated with a database entry (GenBank Features, PubMed Abstracts, Gene Ontology annotation, as well as sequence). Identified keywords are highlighted in bold. The linked number in the column 'Total Hits' links to the complete PSI-BLAST results, where each sequence is annotated with its associated information. PSI-BLAST leads to the raw PSI-BLAST results and GO leads to a tabular listing of the frequency of GO keywords associated with all identified hits.

Using Dip13α/APPL1 as a query ProFAT identified sequence similarity between the first 280 amino acids and BAR-domain containing proteins. The presence of a BAR domain in the N-terminus of Dip13α/APPL1 has been previously reported [3,18].

#### Threading engine

**Figure 4:**
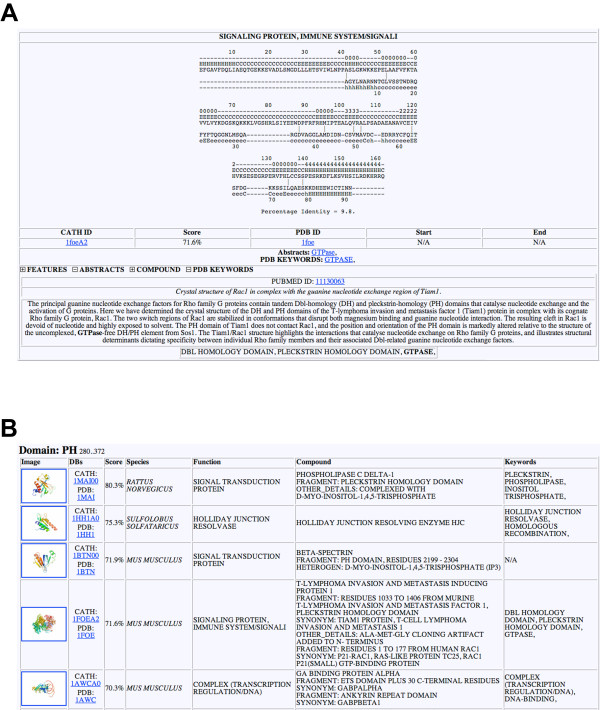
**Typical results from ProFAT's *Threading Engine***. **(A) **Threader alignment of the PH-domain of Dip13α/APPL1 [GenBank:NP_036228]. The *Threading Engine *picked up the crystal structure of the PH-domain of the protein Tiam1 ([PDB:1FOE]). Secondary structure elements are shown above the identified hit. The CATH ID, the threading score, as well as the PDB-ID are given underneath the alignment. The features, abstracts of associated publications, PDB compound information and the PDB-keywords can be individually visualized by the user. In this case, the abstract of the associated paper of 1FOE, as well as the PDB-keywords are shown. **(B) **Processed results of the Threader-output. In this case, the top five hits are shown, including their score, function, compound and keyword information.

The *Threading Engine *performs a Threader run with the selected regions/conserved domains of the input query and subsequently mines information associated with identified structures for keywords from the user-provided keyword list. Information used for text-mining of the *Threading Engine *includes protein databank (PDB-) features, PDB keywords, compound information, as well as literature from PubMed abstracts. The core output of the *Threading Engine *is similar to the *Annotation Engine *described above. By clicking on the number in the column 'Keyword Hits', the user retrieves the alignments provided by Threader 3.5, with individually accessible information concerning the database entry found (Figure 4A). As an example, we show the *Threading Engine *results from the PH-domain of Dip13α/APPL1, which for instance identified the PH-domain of the Rac-GEF Tiam1 ([PDB:1FOE]). The link 'Threader' in the table links to tabular output of the top hits from the Threader search (Figure 4B).

#### HMMerThread

**Figure 5:**
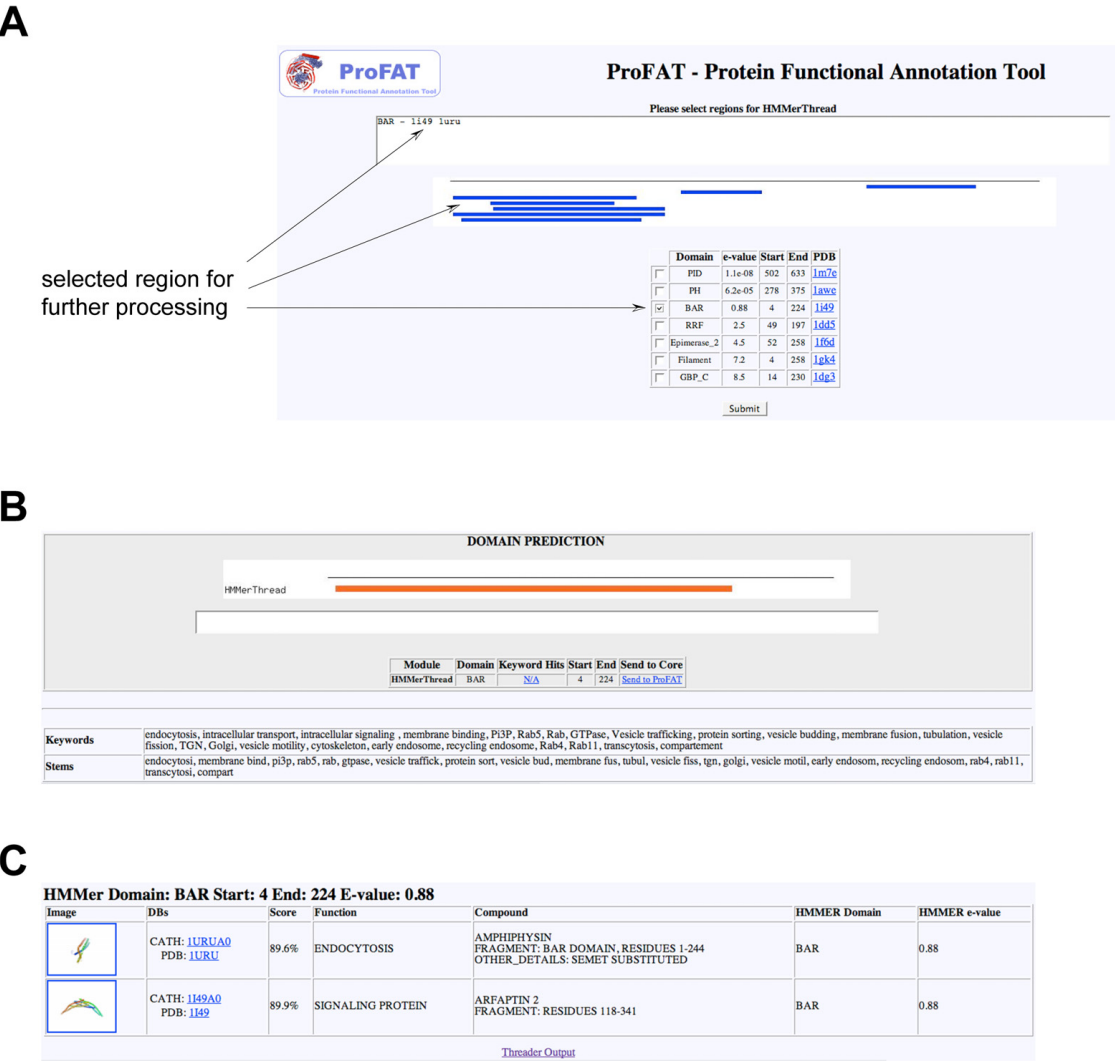
**Typical Output of an *HMMerThread *run**. **(A) **Results from the HMMer-search against the PFAM conserved domain database. The input query was Dip13α/APPL1 [GenBank:NP_036228]. HMMer identified next to the PH- and PTB-domain 5 potential conserved domains in the N-terminus of the protein sequence. Of these five predicted domains, the BAR domain has the lowest E-value of 0.8 and was selected for further processing. **(B) **Results from the threading run identified the BAR domain from residues 4–224. By clicking on the orange bar, the user gets to the detailed results from the threading run (see C). The BAR domain can also be sent to the *Annotation Engine *and *Threading Engine *(link 'Send to ProFAT'). **(C) **Results from the threading run with the predicted N-terminal BAR domain of APPL1. Threader identified the two structures of Amphiphysin ([PDB:1URU]) and Arfaptin2 ([PDB:1I49]), which are both members of the BAR domain family with nearly 90% confidence.

One of the limitations of Threader 3.5 is that its sensitivity drops if the protein region submitted does not correspond in length to the sequence of the crystal structure in the threading database. Threader was for instance not able to detect the BAR-domain in the N-terminal region (amino acids 1 – 280) of Dip13α/APPL1 with a significant score, since the region encompassing the BAR-domain is smaller than the un-annotated N-terminus of the protein. To circumvent this problem we combine an HMMer-based domain search on the input query with a subsequent fold recognition run. Only domains with an associated 3D-structure are considered for further structure prediction (see Figure 1B). When an HMMer search was combined with a threading run for Dip13α/APPL1, two structures of BAR domains were detected as top hits in the threading run (Figure 5). HMMer detected a BAR-domain with an E-value of 0.88 in the N-terminus of Dip13α/APPL1 (Figure 5A, amino acids 4 – 224). When this region was selected for further processing using Threader, it identified the BAR-domains of Amphiphysin and Arfaptin2 with nearly 90% certainty (Figure 5B and 5C). *HMMerThread *could therefore confirm the findings of the *Annotation Engine*.

#### Gene Ontology tree mapping

One limitation of ProFAT is that if the keyword list does not correspond to the actual biological background of the protein input query, results may be misleading. To avoid this, ProFAT maps the GO-annotation of identified hits onto the GO-tree, whereby the number of hits in a certain branch are shown next to the biological processes, molecular functions and cellular compartments. When the user does not find any significant hit with the keyword list used, the ProFAT search can be repeated with a selection of keywords based on the biological function most relevant to the input query, as defined by the associated GO-terms.

#### Design of keyword lists

Text-mining for the selection of biologically relevant hits in ProFAT is performed using keywords from a user-provided list. The results from a ProFAT search are therefore directly influenced by the keywords a user provides for the ProFAT search. While the stemming algorithm [14] used here takes care of differential suffixes of words, users should still follow a few rules in order to obtain optimal results: 1) the user should try to fully describe the process of interest in the keyword list. A CH domain, for example, has been annotated for actin-binding proteins, but is also found in microtubule-associated or cytoskeleton interacting proteins. Assuming that a protein query has been implicated in actin binding, interesting results could therefore be missed, in the case where only the keywords 'actin binding' were present in the keyword list. This is mainly due to firstly, that the actin-binding domain could show remote similarity to a domain which was initially annotated as a microtubule-interacting domain and secondly, because annotations, whether they are manual or automatic, can be inaccurate; 2) the user should try to avoid common words that are found in any GenBank record, like '*RNA*' or '*protein*' or also names of organisms. Other common words found in protein names are for instance '*alpha*', '*beta*' or '*delta*', which should also be avoided; 3) in case the user is uncertain about the exact wording of keywords that describe a certain process, we would recommend to use commonly used wordings as are for instance found in functional annotation databases such as Gene Ontology or the Panther database [19]; 4) if the user already has an idea concerning the identity of a weakly conserved domain found in the protein query, it is recommended to include the name of the domain in the keyword list, as the *Annotation *and *Threading Engines *will then also specifically show those hits that contain similarity to this conserved domain.

### Validation of the ProFAT server

**Figure 6:**
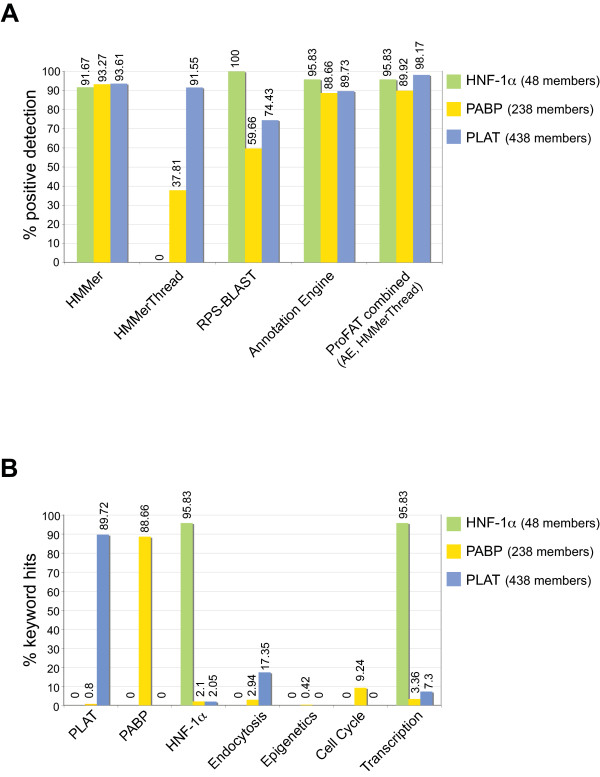
**Evaluation of ProFAT using the domain families PABP, PLAT and HNF-1α**. **(A) **Positive identification of PABP, PLAT and HNF-1α domain family members using *HMMerThread *and the *Annotation Engine*. Based on the Superfamily database [20], all members of the PABP, PLAT and HNF-1α family were subjected to high-throughput *HMMerThread *and *Annotation Engine *searches. Results show the percentage positive identification of family members using these two different pipelines, as well as the domain search programs HMMer and RPS-BLAST. **(B) **Keyword-positive hits of PABP, PLAT and HNF-1α domain family members in ProFAT's *Annotation Engine*. Results show the frequency of keywords identified within the different keyword lists used. Abbreviations used in (A): AE: *Annotation Engine*.

In order to evaluate the performance of the ProFAT server, we chose three domain families from the Superfamily database [20], namely the PABP, PLAT and HNF-1α families. All members of these three domain families, including predicted hits that show only weak sequence conservation, were submitted to automated ProFAT searches using the *Annotation Engine*, as well as *HMMerThread*. The *Annotation Engine *was executed using domain-specific, as well as unspecific keyword lists [see Additional file 1]. Proteins that were correctly predicted using *HMMerThread *searches or the *Annotation Engine*, respectively, were scored (Figure 6). The correct prediction of superfamily association with *HMMerThread *was strongly dependent on the domain (Figure 6A and [see Additional file 2]). While *HMMerThread *correctly identified 92% of all PLAT family members, it detected only 38% of PABP domains and did not find significant scoring for any HNF-1α domain. The failure of *HMMerThread *to detect any HNF-1α also did not change upon increase of *HMMerThread *extensions and increasing the depth of *HMMerThread *hits to 75. A correct domain prediction using fold recognition techniques therefore seems to rely heavily on the domain under analysis, which has been reported before [21]. However, HMMer itself already identified nearly all domains correctly (93% of all 238 PABP family members, 94% of 438 PLAT domains and 92% of 48 HNF-1α domains). RPS-BLAST on the other hand performed worse in terms of domain prediction, with only 60% of correctly predicted PABP domains and 74% correctly predicted PLAT domains. An HNF-1α domain was however detected for all 48 superfamily members by RPS-BLAST, even for more divergent members. The *Annotation Engine *showed an overall good performance on the accurate assignment of superfamilies [see also Additional file 2]. It correctly scored for around 90% of all superfamily members for all three domains in their respective keyword lists (96% of HNF-1α members, 89% of PABP members and 90% of PLAT family members, respectively). Interestingly, the *Annotation Engine *detected almost all weakly conserved members of the PLAT and HNF-1α superfamilies, while it only detected a single predicted protein from the PABP superfamily [see Additional file 2], which suggests that the sequence conservation of predicted PABP-members seems to be too low for detection by PSI-BLAST. A detailed analysis of keyword hits of ProFAT's *Annotation Engine *in all keyword lists is shown in Figure 6B. We observed only a minor false-positive assignment of superfamily members in unrelated keyword lists. Members of the transcription factor family HNF-1α naturally scored with a similar rate in the keyword list '*Transcription*' compared to the keyword list which was designed for '*HNF-1α *' specifically. 17% of the members of the PLAT superfamily, which is a domain characteristic for membrane- and lipid-associated proteins, showed also significant scoring in the keyword list for '*Endocytosis*', which can be explained by the fact that association with membrane or lipids plays an essential role in intracellular transport. The term '*membrane binding*' was furthermore also present in the keyword list designed for '*Endocytosis*' [see Additional file 1]. Finally, the PABP domain is found in the C-terminal region of poly(A)-binding protein, 9% of which seem to score significantly in the keyword list '*Cell Cycle*'. No co-occurring keywords could in this case account for cross-scoring of PABP-family members in '*Cell Cycle*'. However, translational control via poly(A)-binding proteins has also been implicated in the regulation of cell cycle, especially in oocyte maturation (see for instance [22-25]), which could explain the observed hit frequency of PABP superfamily members in '*Cell Cycle*'. These data suggest that ProFAT is indeed able to mine functional annotation of proteins in a highly specific manner. When combining the results of the *Annotation Engine *and *HMMerThread *on the three domain families, ProFAT could detect 96% of all HNF-1α-, 90% of PABP- and 98% of all PLAT family members (Figure 6A, 'ProFAT combined'). Combination of the data from the *Annotation Engine *and *HMMerThread *therefore provides overall better identification than any of the methods alone.

### Identification of novel and weak domain hits using ProFAT

#### Identification of a CH domain in Hook proteins and the microtubule-associated protein KPL2

**Figure 7:**
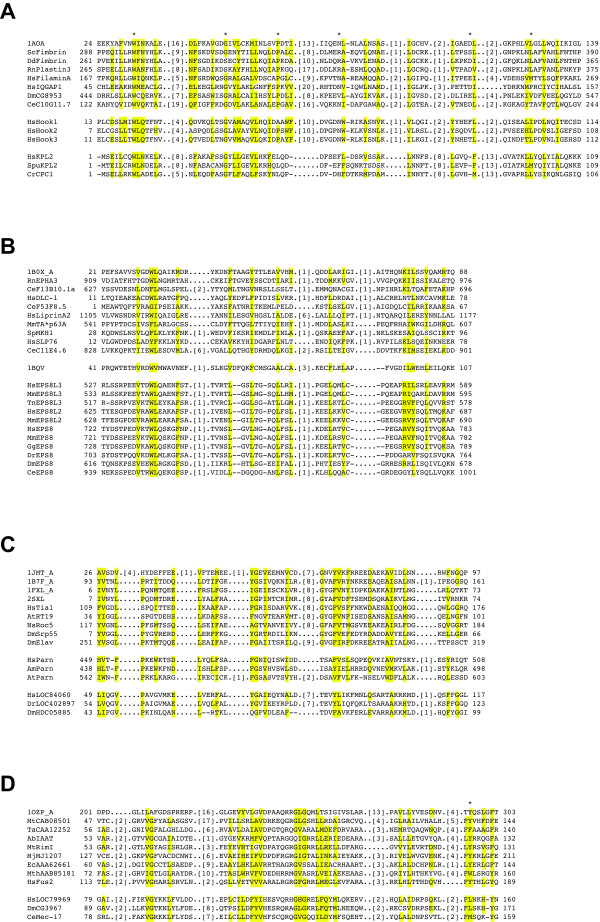
**multiple sequence alignments of weakly conserved domains**. **(A) **Multiple sequence alignment of CH domain family members with human Hook1, Hook2 and Hook3, as well as KPL2 from human, sea urchin and *C. reinhardtii*. Conserved residues are highlighted in yellow, essential residues are marked with an asterix. **(B) **Multiple sequence alignment of Eps8 family members with representatives of the SAM domain family, with conserved residues highlighted in yellow. **(C) **Multiple Sequence alignment of representatives of the RRM domain family with members of the PARN family, as well as human unknown protein LOC84060 and its orthologues from zebrafish and fly. Conserved residues are highlighted in yellow. **(D) **Multiple sequence alignment of human, fly and worm orthologues of unknown protein LOC79969 with representatives of the acetyltransf_1 domain family. Conserved residues are highlighted in yellow, the catalytically important Tyrosine is marked with an asterix. For accession numbers of all proteins shown in alignments **A **– **D**, see [Additional file 10].

Hook3 is a member of the Hook family of proteins involved in intracellular trafficking and associates with its N-terminus to the cytoskeleton [26]. We were interested whether ProFAT would detect sequence similarity with any other protein known to bind to microtubules in the N-terminal part of Hook3 (residues 1 to 153). The *Annotation Engine *identified two Fimbrin-like proteins from *A. thaliana *as potential weak homologues with E-values of 0.96 and 1.1, respectively ([see Additional file 3B]; for figure legends for Additional files 3 to 8 [see Additional file 9]); for accession numbers of proteins used for ProFAT searches and for construction of multiple sequence alignments [see Additional file 10]. The *Threading Engine *did not pick up significant or biologically relevant hits. We therefore submitted Hook3 (1–153) to the *HMMerThread *module, which identified the Calponin Homology (CH) domains from T-Fimbrin ([PDB:1AOA]) and from the APC-binding protein EB1 ([PDB:1PA7], [see Additional file 3C]). We then aligned the three human members of the Hook family to representatives of the CH domain family. As is shown in Figure 7A, all except for one of the essential residues conserved in CH domains are also present in the three human Hook-proteins. The structure of the N-terminus of mouse Hook1 ([PDB:1WIX], Ohashi, et al., unpublished) was recently added to the PDB-database. We compared the structure of the Hook1 CH domain to the CH domain of EB1 using the DALI-server, which gave a Z-score of 8. When 1WIX was used to search the structure database for structural neighbors, it identified the CH domain of Calponin alpha as the first hit with a Z-score of 9.1. The N-terminal domain of Hook1 can therefore be considered to be significantly similar in fold to CH domains. ProFAT was therefore able to correctly identify the N-terminal domain of Hook3 as a CH domain.

KPL2 is an essential component of the central pair complex in ciliated cells. The orthologue from rat was characterized as a gene that is specifically expressed in ciliated cells [27]. The orthologue in *Sus scorfa *was recently linked to an autosomal recessive disease in pigs that leads to immotile short-tail sperm [28]. The orthologue of KPL2 in *Chlamydomonas reinhardtii*, Cpc1, was identified as a component of the central pair complex, which is a large protein complex that regulates the activity of axonemal dynein [29]. The central pair complex consists of 2 central microtubules that associate with a large number of additional factors [30], some of which link the two central microtubules. Central pair complex (CPC-) associated proteins also extrude from this structure and thus help in the assembly of a cylindrical cage of filaments surrounding the microtubules. At open positions in this cage, some CPC-associated proteins interact with external radial spokes and thereby transmit signals that regulate dynein activity for coordinated movement of flagella. Mutations in Cpc1 disrupt the assembly of the central pair complex and alter flagellar beat frequency in *Chlamydomonas *[29]. Biochemical analysis showed that when Cpc1 is deleted, a large portion of the central pair complex is missing.

Rat KPL2 was predicted to have a N-terminal CH domain, with which it could interact with the cytoskeleton or the central microtubule pair [27]. This domain however is undetectable by RPS-BLAST and comes with an insignificant E-value in SMART analysis. We were interested in whether ProFAT would detect a CH domain in human KPL2. The domain search of ProFAT detected a domain of unknown function DUF1042 in the N-terminal part of the protein, which was selected for further processing [see Additional file 4A]. HMMer, on the other hand, detected the presence of a CH domain between amino acids 1 – 105 in the sequence, which was sent to Threader [see Additional file 4A]. The *Annotation Engine *of ProFAT identified among other CH domain – containing sequences, the proteins Mal3 from *S. pombe *and the microtubule-associated protein EB1 from *Arabidopsis *[see Additional file 4B]. Along the same lines, *HMMerThread *detected the presence of a CH domain with 83% confidence [see Additional file 4C]. The alignment of 3 KPL2 orthologues with representatives from the CH domain family reveals good conservation of KPL2 to CH domain family members (Figure 7A). These results suggest that the domain DUF1042 is essentially a member of the CH domain family.

#### Identification of a SAM domain in the C-terminus of EPS8 family members

Eps8 proteins are downstream targets of the Epidermal Growth Factor (EGF) pathway. Members of this protein family are implicated in EGF-mediated signal transduction, though their exact role is so far unknown. It has been shown that Eps8 coordinates EGF-receptor signaling via regulation of small GTPases. A C-terminal effector region in Eps8, for instance regulates activation of Rac, which leads to actin cytoskeleton remodeling [31]. Eps8 family proteins are predicted to have a SAM domain in the C-terminus of the protein [31]. Domain searches using RPS-BLAST and/or SMART fail to identify this domain, even at permissive E-values. We were interested in whether ProFAT could detect the SAM domain in those proteins. The domain search of ProFAT identified an EPS8/PTB domain in the N-terminus of EPS8L3, as well as a SH3 domain in the C-terminal part, but failed to recognize the SAM domain. HMMer on the other hand detected a SAM_1 domain with an E-value of 2 in the C-terminus of the protein, which was selected for further processing [see Additional file 5A]. ProFAT's *Annotation Engine *detected SAM-domain containing proteins, as, for instance, a sequence from chicken and the kinase suppressor of Ras from *Drosophila simulans *[see Additional file 5B]. The *HMMerThread *pipeline predicted a SAM_1 domain in the C-terminus of EPS8L3 with a certainty of over 90% [see Additional file 5C]. The multiple sequence alignment of Eps8 and Eps8-like proteins 2 and 3 with representatives of the SAM domain family, as well as the structural representative of the SAM_PNT domain, which is a subfamily of the SAM domain, shows a conserved pattern of hydrophobic, aromatic and charged amino acids (Figure 7B). These results suggest that the C-termini of Eps8 and Eps8 like proteins contain a SAM domain, as was proposed previously [31].

#### Identification of an RRM domain in PARN proteins and an uncharacterized protein family

The poly(A)-specific ribonuclease PARN is a 3' exonuclease which is involved in the destruction of cellular mRNAs [32]. Members of the PARN family contain a split CAF1 domain, which has ribonuclease catalytic activity. In the center of the CAF1 domain, RPS-BLAST predicts a PARN_R3H domain, which is predicted to bind single- or double-stranded RNAs. RPS-BLAST also predicts a weakly conserved RRM domain C-terminal of the CAF1 domain with an E-value of 1.8. We were interested as to whether ProFAT could detect the weakly conserved RRM domain in human PARN. The domain search of ProFAT correctly predicts the CAF1 and PARN_RH3 domains and the HMMer module of *HMMerThread *predicts the presence of an RRM_1 domain adjacent to the CAF1 domain [see Additional file 6A]. We selected the RRM_1 module for further processing with *HMMerThread*, as well as the C-terminal part of PARN for analysis using the *Annotation *and *Threading Engines *of ProFAT. The *Annotation Engine *identified, as an example, the Bruno-like RNA binding protein 5 from chicken [see Additional file 6B]. *HMMerThread *identified RRM motifs from several crystallized proteins with a confidence of nearly 90% [see Additional file 6C]. The crystal structure of the region containing the RRM domain of a PARN family member has been determined (Nagata T., et al., 2004, unpublished; [PDB:1WHV]). Using the DALI server, we searched for similar structures to 1WHV. The closest hit is the structure of the central RRM of human La protein ([PDB:1S79], [33]), which is detected with a significant score of 7.5. We next performed a multiple sequence alignment of PARN family members to representatives of the RRM domain (Figure 7C) and observe a high level of conservation between these two domains. ProFAT was therefore able to detect the weakly conserved RRM domain in PARN family members.

The uncharacterized human protein LOC84060 has not been associated with any biological function. Domain searches using standard parameters did not reveal any conserved domains for this protein. However, when increasing the E-value in the RPS-BLAST search, an RRM domain is found with an E-value of 4.6. Assuming that this protein would be involved in RNA metabolism or regulation, we submitted the protein sequence of LOC84060 to the ProFAT server. The domain search pipeline of ProFAT did not find any conserved domain, while HMMer identified the presence of an RRM_1 domain in this protein [see Additional file 7A]. We selected the RRM_1 domain for processing with *HMMerThread *and submitted the protein sequence of LOC84060 to ProFAT's *Annotation *and *Threading Engine*. For more accurate results, we invoked the option of splitting the input sequence using 150 amino acids. ProFAT's *Annotation Engine *identified among others the RRM domain in the poly (A)-binding protein PABPC from human [see Additional file 7B]. *HMMerThread *found the RRM_1 domain of splicing factor U2AF as significantly similar [see Additional file 7C]. Next we aligned LOC84060 to representatives of the RRM domain (Figure 7C). The multiple sequence alignment reveals that LOC84060 shares all except for two residues that are conserved in this domain family. Based on this data, we suggest that LOC84060 is a RRM domain containing, RNA-binding protein.

#### Identification of an acetyltransferase domain in the unknown human protein LOC79969

No functional information is so far available for the uncharacterized human protein LOC79969. Domain searches using RPS-BLAST or SMART predict the presence of a domain of unknown function, DUF738. As there was no hint on the biological context this protein could be associated with, we performed only an *HMMerThread *search with the protein sequence of LOC79969. HMMer detected a weakly conserved acetyltransferase domain within the DUF738 region [see Additional file 8A]. We selected the predicted Acetyltransf_1 domain for further processing using the threading pipeline of *HMMerThread*, which identified the 3-dimensional structures of several acetyltransferases with a confidence of nearly 90% [see Additional file 8B]. We next aligned members of the LOC79969 family to representatives of the Acetyltransf_1 domain family (Figure 7D). LOC79969 seems to be most closely related to the GNAT subfamily of acetyltransferases. Interestingly, the proposed catalytic Tyrosine residue at the C-terminus of the Acetyltranferase domain (reviewed in [34]) is mutated to a Leucine in human and fly LOC79969 and a Methionine in *C. elegans*. A conserved Tyrosine is however located 4 residues C-terminal to the proposed catalytic site. As our data suggest that LOC79969 adopts a GNAT-like fold, it will have to be tested experimentally, whether the Acetyltransf_1 domain is catalytically active.

### Applications of ProFAT

ProFAT finds its utility in several applications: 1) the ProFAT server should be used when standard similarity search programs fail to predict the function of a so-far uncharacterized protein that can be associated with a certain cellular process/molecular function. In this case, ProFAT would be used as an aid for post-filtering of complex Threading and PSI-BLAST outputs; 2) the user might be interested in whether a conserved domain shows remote sequence similarity or is structurally related to proteins from a specific cellular process/molecular function and can therefore use ProFAT to specifically search for weakly related sequences or structures that are found in the biological context of interest; 3) the domain prediction pipeline is applicable to regions of proteins with no obvious conserved domain. In this case, the combination of RPS-BLAST and a subsequent BLAST-search of weak domain hits with a text-mining step can strengthen evidence from subtle sequence similarity with additional biologically relevant evidence; 4) finally, *HMMerThread *presents itself as a very powerful pipeline for accurate prediction of weakly conserved domains by looking for remote sequence similarity with conserved domain hits in combination with a subsequent threading step. *HMMerThread *in addition has the advantage of not relying on the user-provided keyword list and can be applied to proteins, which cannot be associated with any biological function. This module can therefore be used as a means of predicting weakly conserved domains with high accuracy.

## Conclusion

ProFAT is a powerful tool for the uncovering of remote but biologically relevant relationships between sequences. While highly powerful tools are already available to discover subtle sequence similarity, for example profile-based database search methods and fold recognition techniques, only few methods so far exist that also provide a means to combine these search tools with a literature-mining step. In particular the text-mining of associated literature abstracts makes ProFAT unique in post-filtering database sequences based on biological features found in associated primary literature. While tools such as OntoBLAST and SAWTED use secondary annotation of sequences for post-filtering of database search results, ProFAT goes back to primary published information of sequence entries, which helps to circumvent the problem of sometimes error-prone functional information found in the annotation of sequences. The strength of ProFAT furthermore lies in the combination of sequence- and structure-based search tools that are able to reliably detect weak sequence relationships. Finally, ProFAT is highly flexible and allows the user to tailor a database search to his own biological interest.

## Availability and requirements

• **Program name: **ProFAT

• **Project home page: **

• **Operating Systems: **platform independent

• **Programming language: **Perl

• **other requirements: **Web-browser, valid e-mail address

• **License: **GNU public license

• **Any restrictions to use by non-academics: **Commercial users are not able to use *HMMerThread *or the *Threading Engine *due to license restrictions for Threader 3.5

## Authors' contributions

CRB is responsible for the scripting and implementation of most parts of the ProFAT web-server. He also designed and executed the high-throughput ProFAT pipeline for large-scale searches and applied the ProFAT server to the functional annotation of proteins mentioned in this manuscript.

VS has helped with the design of the ProFAT web-server. He also has scripted the tool for the mapping of hits on GO-terms in the Gene Ontology tree and has helped with statistical evaluation of significant hits from high-throughput ProFAT searches.

BH conceived of and supervised this study and helped with the design and testing of the ProFAT web-server. She participated in the alignment of the remotely conserved domains mentioned here, analyzed results from ProFAT's large-scale results and was drafting this manuscript.

All authors read and approved the final manuscript.

## Supplementary Material

Additional File 1 Table containing all keyword lists used within this study. Click here for file

Additional File 2 Detailed results of bulk searches using the *Annotation Engine *and *HMMerThread *for all members of the HNF-1α, PABP and PLAT superfamilies. Click here for file

Additional File 3 Original ProFAT results for human protein Hook3, which was predicted as related to the CH domain family. Click here for file

Additional File 4 ProFAT results for KPL2, which was predicted as related to the CH domain family. Click here for file

Additional File 5 Original ProFAT results for EPS8L3 protein, resulting in the detection of a SAM domain in the C-terminus of the protein. Click here for file

Additional File 6 Original ProFAT results for human protein PARN which has a weakly conserved RRM domain. Click here for file

Additional File 7 ProFAT result for human protein LOC84060, which is predicted to have a weakly conserved RRM domain. Click here for file

Additional File 8 Original ProFAT results for the human protein LOC79969, which has a predicted acetyltransferase domain. Click here for file

Additional File 9 Figure legends for Additional files 3 to 8. Click here for file

Additional File 10 Table containing all accession numbers used for multiple sequence alignments in Figure 7. A detailed manual of the usage of the ProFAT web server containing screenshots of typical ProFAT results is available online [35]. Click here for file
