# Supplementary material for: ProFAT: a web-based tool for the functional annotation of protein sequences
Source: BMC Bioinformatics. 2006 Oct 23;7:466. doi: 10.1186/1471-2105-7-466 (PMC1636073; doi:10.1186/1471-2105-7-466)
Supplement: Additional File 1 — Table containing all keyword lists used within this study. [file 1471-2105-7-466-S1.pdf]

Additional File 1: Keyword lists used

Cell Cycle	cell cycle<br>APC<br>cyclin<br>cyclin-dependent kinase<br>anaphase promoting complex<br>activator<br>cdc20<br>cdh1<br>fizzy<br>separase<br>cohesin<br>sister chromatid cohesion<br>securin<br>cell division<br>proliferation<br>prophase<br>metaphase<br>anaphase<br>telophase<br>S-phase<br>M-phase<br>interphase<br>replication<br>S-phase promoting complex<br>meiosis<br>mitosis<br>spindle pole<br>centrosome<br>centromere<br>spindle<br>metaphase plate<br>ubiquitin ligase<br>cell-cycle dependent proteolysis<br>bipolar attachement<br>monopolar attachement<br>cytokinesis
Endocytosis	endocytosis<br>intracellular transport<br>intracellular signaling<br>membrane binding<br>Pi3P<br>Rab5<br>Rab<br>GTPase<br>Vesicle trafficking<br>protein sorting<br>vesicle budding<br>membrane fusion<br>tubulation<br>vesicle fission<br>TGN<br>Golgi<br>vesicle motility<br>cytoskeleton<br>early endosome

	recycling endosome<br>Rab4<br>Rab11<br>transcytosis<br>compartement
Epigenetics	epigenetics<br>chromatin<br>histone methylation<br>jumonji domain<br>SET domain<br>LSD1
Transcription	transcriptional control<br>transcriptional regulation<br>RNA polymerase<br>RNAP<br>elongation<br>transcription factor<br>transcriptional termination<br>polII<br>polI<br>polIII<br>enhancer<br>promoter<br>enhancer element<br>DNA-binding<br>TATA box-binding<br>transcriptional repressor<br>transcription initation factor
APPL1	endocytosis<br>intracellular transport<br>vesicle trafficking<br>vesicle movement<br>vesicle fission<br>vesicle fusion<br>vesicle motility<br>tubulation<br>GTPase binding<br>small GTPase<br>Rab<br>cytoskeleton<br>actin binding<br>tubulin binding<br>microtubules<br>actin<br>tubulin<br>signaling
Microtubule/Cytoskeleton-associated proteins	endocytosis<br>vesicle trafficking<br>vesicle movement<br>cytoskeleton<br>actin binding<br>tubulin binding<br>actin<br>tubulin<br>microtubules<br>motor protein

RNA binding proteins	RNA binding<br>nucleic acid binding<br>translation<br>translational regulation<br>3'UTR<br>RNA binding domain<br>RBD<br>RRM<br>poly-adenylation<br>adenlyation<br>RNase<br>RNA degradation<br>RNA stability
EPS8 like proteins	translation<br>transcription<br>nucleic acid binding<br>RNA binding<br>UTR<br>protein interaction<br>homodimerization<br>heterodimerization<br>signaling
PABP domain family	polyadenylate-binding<br>poly-A tail<br>cap structure<br>translation<br>translational regulation<br>activation of translation<br>repression of translation<br>translational activation<br>translational repression<br>RNA binding domain<br>RBD<br>PABP<br>polyadenlyate-binding protein
PLAT domain family	lipid binding<br>lipid association<br>lipid metabolism<br>membrane binding<br>membrane association<br>membrane attachement<br>membrane dynamics<br>polycystin-1<br>lipoxygenase<br>lipoxygenase homology<br>colipase<br>lipogenase<br>lipase
HNF-1α	transcription factor<br>hepatocyte nuclear factor<br>homeo-box<br>DNA-binding<br>regulation of transcription<br>transcriptional regulation<br>transcriptional activation<br>activation of transcription

transcriptional transactivation
transcription
enhancer
enhancer of transcription
promoter sequence
enhancer sequence
