# Supplementary material for: ProFAT: a web-based tool for the functional annotation of protein sequences
Source: BMC Bioinformatics. 2006 Oct 23;7:466. doi: 10.1186/1471-2105-7-466 (PMC1636073; doi:10.1186/1471-2105-7-466)
Supplement: Additional File 3 — Original ProFAT results for human protein Hook3, which was predicted as related to the CH domain family. [file 1471-2105-7-466-S3.pdf]

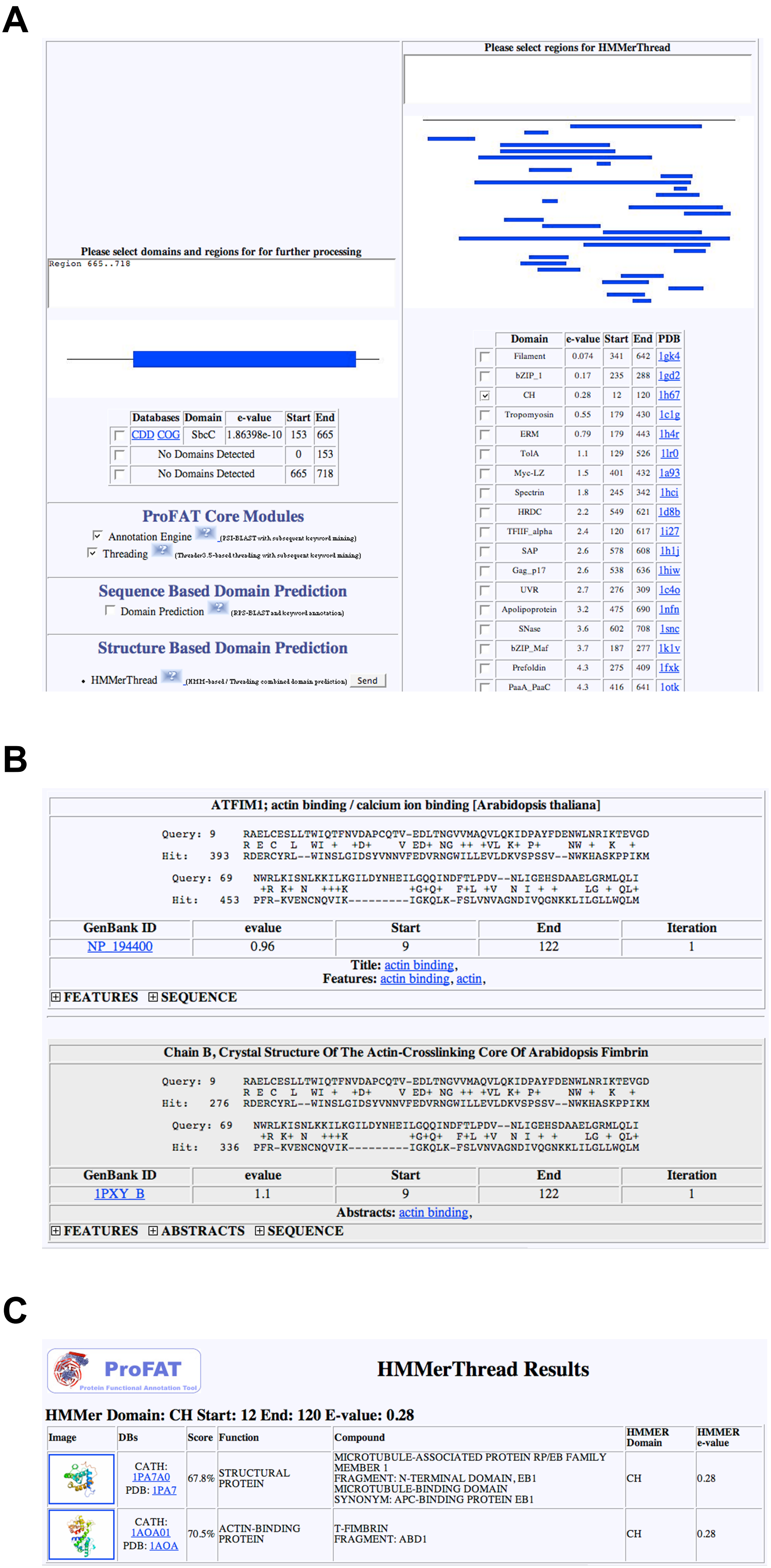
A
Please select regions for HMMerThread
Please select domains and regions for for further processing
Region 665..718
Databases | Domain | e-value | Start | End
CDD COG | SbcC | 1.86398e-10 | 153 | 665
No Domains Detected | 0 | 153
No Domains Detected | 665 | 718
ProFAT Core Modules
Annotation Engine (PSI-BLAST with subsequent keyword mining)
Threading (Threader3.5-based threading with subsequent keyword mining)
Sequence Based Domain Prediction
Domain Prediction (RPS-BLAST and keyword annotation)
Structure Based Domain Prediction
HMMerThread (HMM-based / Threading combined domain prediction) Send
Domain | e-value | Start | End | PDB
Filament | 0.074 | 341 | 642 | 1gk4
bZIP_1 | 0.17 | 235 | 288 | 1gd2
CH | 0.28 | 12 | 120 | 1h67
Tropomyosin | 0.55 | 179 | 430 | 1c1g
ERM | 0.79 | 179 | 443 | 1h4r
TolA | 1.1 | 129 | 526 | 1lr0
Myc-LZ | 1.5 | 401 | 432 | 1a93
Spectrin | 1.8 | 245 | 342 | 1hci
HRDC | 2.2 | 549 | 621 | 1d8b
TFIIF_alpha | 2.4 | 120 | 617 | 1i27
SAP | 2.6 | 578 | 608 | 1h1j
Gag_p17 | 2.6 | 538 | 636 | 1hiw
UVR | 2.7 | 276 | 309 | 1c4o
Apolipoprotein | 3.2 | 475 | 690 | 1nfn
SNase | 3.6 | 602 | 708 | 1snc
bZIP_Maf | 3.7 | 187 | 277 | 1k1v
Prefoldin | 4.3 | 275 | 409 | 1fxk
PaaA_PaaC | 4.3 | 416 | 641 | 1otk
B
ATFIM1; actin binding / calcium ion binding [Arabidopsis thaliana]
Query: 9 RAELCESLLTWIQTFNVDAPCQTV-EDLTNGVVMAQVLQKIDPAYFDENWLNRIKTEVGD
R E C L WI + +D+ V ED+ NG ++ +VL K+ P+ NW + K +
Hit: 393 RDERCYRL--WINSLGIDSYVNNVFEDVRNGWILLEVLDKVSPSSV--NWKHASKPPIKM
Query: 69 NWRLKISNLKKILKGILDYNHEILGQQINDFTLPDV--NLIGEHSDAAELGRMLQLI
+R K+ N +++K +G+Q+ F+L +V N I + + LG + QL+
Hit: 453 PFR-KVENCNQVIK---------IGKQLK-FSLVNVAGNDIVQGNKKLILGLLWQLM
GenBank ID | evalue | Start | End | Iteration
NP_194400 | 0.96 | 9 | 122 | 1
Title: actin binding,
Features: actin binding, actin,
FEATURES SEQUENCE
Chain B, Crystal Structure Of The Actin-Crosslinking Core Of Arabidopsis Fimbrin
Query: 9 RAELCESLLTWIQTFNVDAPCQTV-EDLTNGVVMAQVLQKIDPAYFDENWLNRIKTEVGD
R E C L WI + +D+ V ED+ NG ++ +VL K+ P+ NW + K +
Hit: 276 RDERCYRL--WINSLGIDSYVNNVFEDVRNGWILLEVLDKVSPSSV--NWKHASKPPIKM
Query: 69 NWRLKISNLKKILKGILDYNHEILGQQINDFTLPDV--NLIGEHSDAAELGRMLQLI
+R K+ N +++K +G+Q+ F+L +V N I + + LG + QL+
Hit: 336 PFR-KVENCNQVIK---------IGKQLK-FSLVNVAGNDIVQGNKKLILGLLWQLM
GenBank ID | evalue | Start | End | Iteration
1PXY_B | 1.1 | 9 | 122 | 1
Abstracts: actin binding,
FEATURES ABSTRACTS SEQUENCE
C
ProFAT
Protein Functional Annotation Tool
HMMerThread Results
HMMer Domain: CH Start: 12 End: 120 E-value: 0.28
Image | DBs | Score | Function | Compound | HMMER Domain | HMMER e-value
CATH: 1PA7A0 PDB: 1PA7 | 67.8% | STRUCTURAL PROTEIN | MICROTUBULE-ASSOCIATED PROTEIN RP/EB FAMILY MEMBER 1 FRAGMENT: N-TERMINAL DOMAIN, EB1 MICROTUBULE-BINDING DOMAIN SYNONYM: APC-BINDING PROTEIN EB1 | CH | 0.28
CATH: 1AOA01 PDB: 1AOA | 70.5% | ACTIN-BINDING PROTEIN | T-FIMBRIN FRAGMENT: ABD1 | CH | 0.28
