# Supplementary material for: ProFAT: a web-based tool for the functional annotation of protein sequences
Source: BMC Bioinformatics. 2006 Oct 23;7:466. doi: 10.1186/1471-2105-7-466 (PMC1636073; doi:10.1186/1471-2105-7-466)
Supplement: Additional File 4 — ProFAT results for KPL2, which was predicted as related to the CH domain family. [file 1471-2105-7-466-S4.pdf]

A

Please select domains and regions for for further processing

Region 870..1822

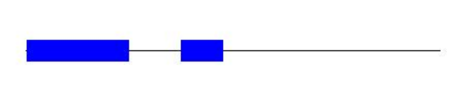

	Databases	Domain	e-value	Start	End
☑	CDD PFAM	DUF1042	3.47876e-40	5	154
☐	CDD COG	Smc	4.72078e-05	158	453
☐	CD	ADK	1.54375e-10	685	870
☐	No Domains Detected			453	685
☐	No Domains Detected			870	1822

ProFAT Core Modules

☑ Annotation Engine ? (PSI-BLAST with subsequent keyword mining)

☑ Threading ? (Threader3.5-based threading with subsequent keyword mining)

Sequence Based Domain Prediction

☐ Domain Prediction ? (RPS-BLAST and keyword annotation)

Structure Based Domain Prediction

- HMMerThread ? (HMM-based / Threading combined domain prediction) Send

Please select regions for HMMerThread

TFIIF_alpha - 1i27 1j2x 1nha 1onv 1f3u

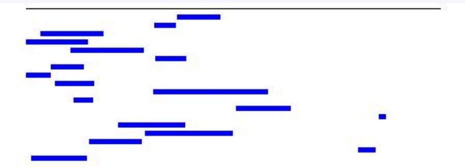

	Domain	e-value	Start	End	PDB
☐	ADK	0.083	668	856	3aky
☐	TBCA	0.67	568	658	1h7c
☐	GBP_C	3	68	339	1dg3
☐	DNA_topoisoIV	3.5	1	269	1ab4
☐	TolA	3.7	199	516	1lr0
☐	cobW	3.9	570	705	1nij
☐	Cullin	4.5	112	252	1ldj
☑	CH	4.7	1	105	1h67
☐	Smg4_UPF3	4.8	132	299	1uw4
☐	TFIIF_alpha	5.1	562	1062	1i27
☐	KE2	5.8	213	296	1fxk
☐	CDK5_activator	6	927	1166	1h4l
☐	efhand	6.3	1556	1584	1g4y
☐	Lyase_8_N	6.5	406	699	1lxk
☐	HSP70	6.6	528	908	1hx1
☐	ERM	6.8	278	510	1h4r
☐	CRS1_YhbY	7.2	1464	1536	1ln4
☐	SBP_bac_9	7.5	24	267	1pq4

Submit

B

mal3 [Schizosaccharomyces pombe]

```
Query: 3    WLNKELKVSRTVSPKSFAKAFSSGYLLGEVLHKFELQDDFS--EFLDSRVSSAKLNNFSR
            W+N+   +  T        +    GY + ++                F          +NN+
Hit:   11   WINQVTSLGLTRI-----EDCGKGYAMIQIFDSIYQDIPLKKVNFE-CNNEYQYINNWKV

                      Query: 63   LEPTLNLLGVQ
                                  L+      G+
                      Hit:   71   LQQVFLKKGID
```

GenBank ID	evalue	Start	End	Iteration
CAA92392	1.4	3	71	3

Features: microtubule,

⊟ FEATURES ⊞ ABSTRACTS ⊞ SEQUENCE

Protein>	1..308	name	mal3
CDS>	1..308	coded_by	join(Z68198.1:35734..35775, Z68198.1:35836..36720)
CDS>	1..308	db_xref	GOA:Q10113
CDS>	1..308	db_xref	InterPro:IPR001715
CDS>	1..308	db_xref	InterPro:IPR004953
CDS>	1..308	db_xref	UniProt/Swiss-Prot:Q10113
CDS>	1..308	gene	mal3
CDS>	1..308	note	EB1 domain; calponin homology domain; involved in **microtubule** organization (PMID 9348288); involved in spindle formation (PMID 11102508); similar to S. cerevisiae YER016W

putative microtubule-associated protein EB1 [Arabidopsis thaliana]

```
Query: 3    WLNKELKVSRTVSPKSFAKAFSSGYLLGEVLH-KFELQDDFS--EFLDSRVSSAKLNNFS
            W+N  L ++ +        +  +SG +  ++L   F          F         + N+
Hit:   22   WINDRLHLNLSRI-----EEAASGAVQCQMLDMTFPGVVPMHKVNFEAKNEYE-MIQNYK

                 Query: 63   RLEPTLNLLGVQFDQNVAHGIITEKP
                               ++      L +     V + ++  +P
                 Hit:   82   VMQEVFTKLKITKPLEV-NRLVKGRP
```

GenBank ID	evalue	Start	End	Iteration
BAC42296	1.4	3	85	4

Title: microtubul,
Features: microtubul,

⊞ FEATURES ⊞ SEQUENCE

C

HMMer Domain: CH Start: 1 End: 105 E-value: 4.7

Image	DBs	Score	Function	Compound	HMMER Domain	HMMER e-value
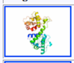	CATH: 1AOA01<br>PDB: 1AOA	82.8%	ACTIN-BINDING PROTEIN	T-FIMBRIN<br>FRAGMENT: ABD1	CH	4.7
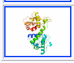	CATH: 1AOA02<br>PDB: 1AOA	79.0%	ACTIN-BINDING PROTEIN	T-FIMBRIN<br>FRAGMENT: ABD1	CH	4.7

Threader Output
