# Supplementary material for: ProFAT: a web-based tool for the functional annotation of protein sequences
Source: BMC Bioinformatics. 2006 Oct 23;7:466. doi: 10.1186/1471-2105-7-466 (PMC1636073; doi:10.1186/1471-2105-7-466)
Supplement: Additional File 5 — Original ProFAT results for EPS8L3 protein, resulting in the detection of a SAM domain in the C-terminus of the protein. [file 1471-2105-7-466-S5.pdf]

A

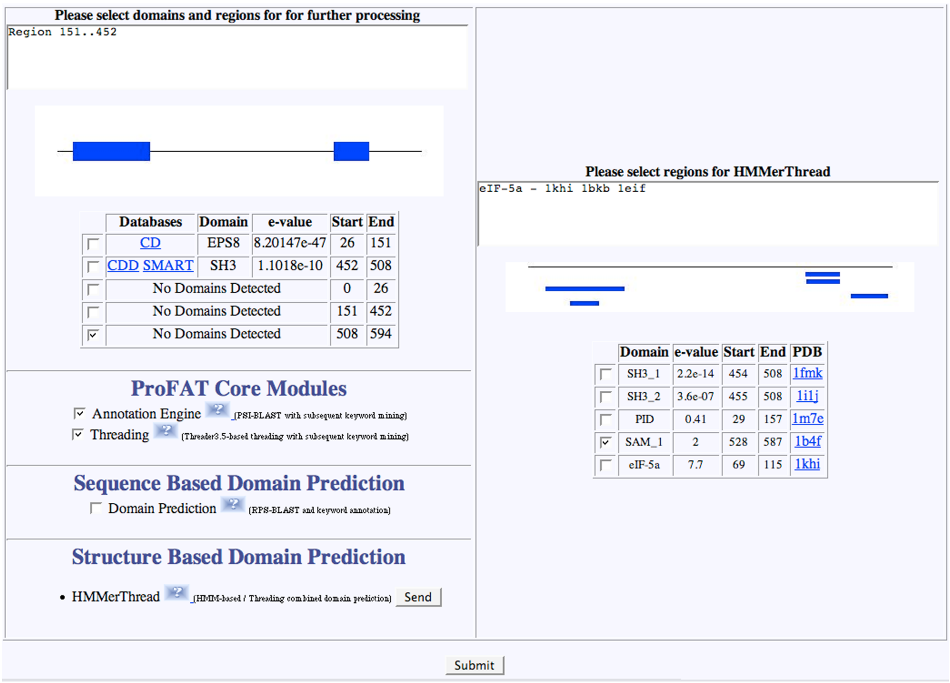
Please select domains and regions for for further processing
Region 151..452
Databases Domain e-value Start End
CD EPS8 8.20147e-47 26 151
CDD SMART SH3 1.1018e-10 452 508
No Domains Detected 0 26
No Domains Detected 151 452
No Domains Detected 508 594
ProFAT Core Modules
Annotation Engine (PSI-BLAST with subsequent keyword mining)
Threading (Threader3.5-based threading with subsequent keyword mining)
Sequence Based Domain Prediction
Domain Prediction (RPS-BLAST and keyword annotation)
Structure Based Domain Prediction
HMMerThread (HMM-based / Threading combined domain prediction)
Send
Please select regions for HMMerThread
eIF-5a - 1khi 1bkb 1eif
Domain e-value Start End PDB
SH3_1 2.2e-14 454 508 1fmk
SH3_2 3.6e-07 455 508 1i1j
PID 0.41 29 157 1m7e
SAM_1 2 528 587 1b4f
eIF-5a 7.7 69 115 1khi
Submit

B

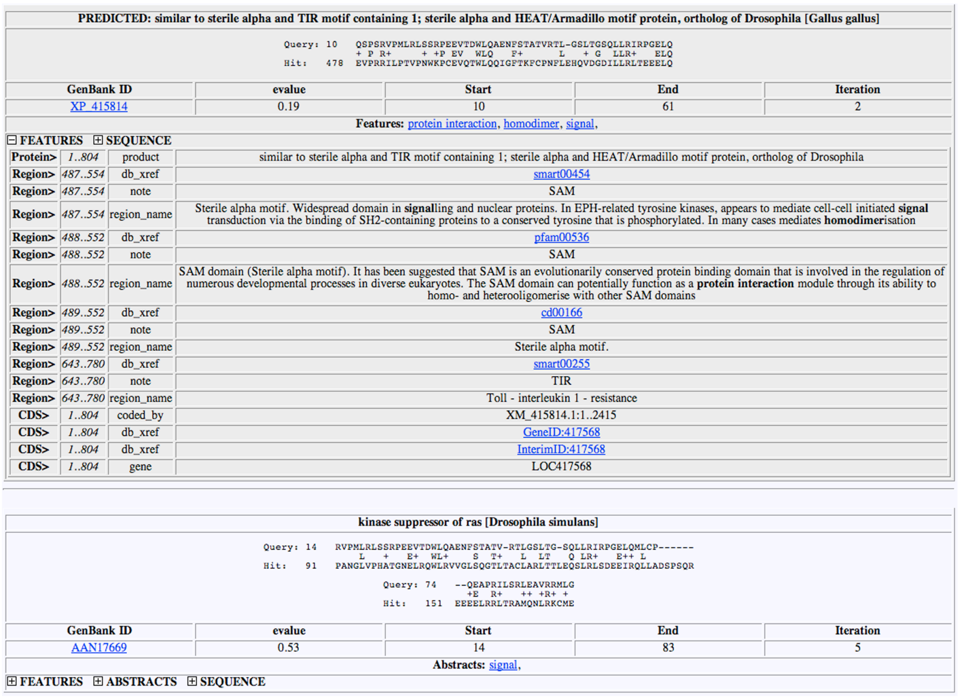
PREDICTED: similar to sterile alpha and TIR motif containing 1; sterile alpha and HEAT/Armadillo motif protein, ortholog of Drosophila [Gallus gallus]
Query: 10 QSPSRVPMLRLSSRPEEVTDWLQAENFSTATVRTL-GSLTGSQLLRIRPGELQ
+ P R+ + +P EV WLQ F+ L + G LLR+ ELQ
Hit: 478 EVPRRILPTVPNWKPCEVQTWLQQIGFTKFCPNFLEHQVDGDILLRLTEEELQ
GenBank ID evalue Start End Iteration
XP_415814 0.19 10 61 2
Features: protein interaction, homodimer, signal,
FEATURES SEQUENCE
Protein> 1..804 product similar to sterile alpha and TIR motif containing 1; sterile alpha and HEAT/Armadillo motif protein, ortholog of Drosophila
Region> 487..554 db_xref smart00454
Region> 487..554 note SAM
Region> 487..554 region_name Sterile alpha motif. Widespread domain in signalling and nuclear proteins. In EPH-related tyrosine kinases, appears to mediate cell-cell initiated signal transduction via the binding of SH2-containing proteins to a conserved tyrosine that is phosphorylated. In many cases mediates homodimerisation
Region> 488..552 db_xref pfam00536
Region> 488..552 note SAM
Region> 488..552 region_name SAM domain (Sterile alpha motif). It has been suggested that SAM is an evolutionarily conserved protein binding domain that is involved in the regulation of numerous developmental processes in diverse eukaryotes. The SAM domain can potentially function as a protein interaction module through its ability to homo- and heterooligomerise with other SAM domains
Region> 489..552 db_xref cd00166
Region> 489..552 note SAM
Region> 489..552 region_name Sterile alpha motif.
Region> 643..780 db_xref smart00255
Region> 643..780 note TIR
Region> 643..780 region_name Toll - interleukin 1 - resistance
CDS> 1..804 coded_by XM_415814.1:1..2415
CDS> 1..804 db_xref GeneID:417568
CDS> 1..804 db_xref InterimID:417568
CDS> 1..804 gene LOC417568
kinase suppressor of ras [Drosophila simulans]
Query: 14 RVPMLRLSSRPEEVTDWLQAENFSTATV-RTLGSLTG-SQLLRIRPGELQMLCP------
L + E+ WL+ S T+ L LT Q LR+ E++ L
Hit: 91 PANGLVPHATGNELRQWLRVVGLSQGTLTACLARLTTLEQSLRLSDEEIRQLLADSPSQR
Query: 74 --QEAPRILSRLEAVRRMLG
+E R+ ++ +R+ +
Hit: 151 EEEELRRLTRAMQNLRKCME
GenBank ID evalue Start End Iteration
AAN17669 0.53 14 83 5
Abstracts: signal,
FEATURES ABSTRACTS SEQUENCE

C

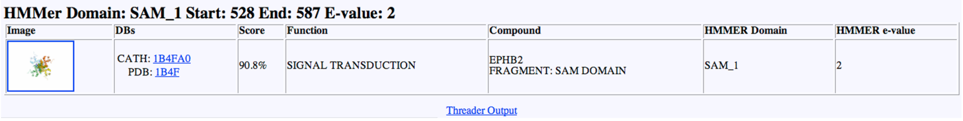
HMMer Domain: SAM_1 Start: 528 End: 587 E-value: 2
Image DBs Score Function Compound HMMER Domain HMMER e-value
CATH: 1B4FA0 PDB: 1B4F 90.8% SIGNAL TRANSDUCTION EPHB2 FRAGMENT: SAM DOMAIN SAM_1 2
Threader Output
