# Supplementary material for: ProFAT: a web-based tool for the functional annotation of protein sequences
Source: BMC Bioinformatics. 2006 Oct 23;7:466. doi: 10.1186/1471-2105-7-466 (PMC1636073; doi:10.1186/1471-2105-7-466)
Supplement: Additional File 6 — Original ProFAT results for human protein PARN which has a weakly conserved RRM domain. [file 1471-2105-7-466-S6.pdf]

A

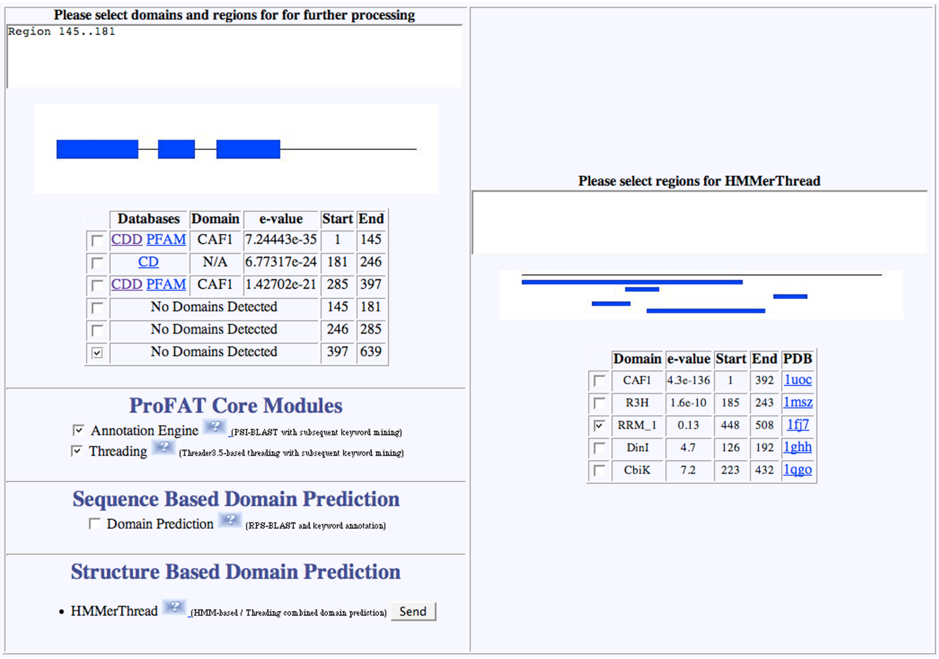

Please select domains and regions for for further processing

Region 145..181

	Databases	Domain	e-value	Start	End
☐	CDD PFAM	CAF1	7.24443e-35	1	145
☐	CD	N/A	6.77317e-24	181	246
☐	CDD PFAM	CAF1	1.42702e-21	285	397
☐	No Domains Detected			145	181
☐	No Domains Detected			246	285
☑	No Domains Detected			397	639

ProFAT Core Modules

☑ Annotation Engine (PSI-BLAST with subsequent keyword mining)

☑ Threading (Threader3.5-based threading with subsequent keyword mining)

Sequence Based Domain Prediction

☐ Domain Prediction (RPS-BLAST and keyword annotation)

Structure Based Domain Prediction

- HMMerThread (HMM-based / Threading combined domain prediction) Send

Please select regions for HMMerThread

	Domain	e-value	Start	End	PDB
☐	CAF1	4.3e-136	1	392	1uoc
☐	R3H	1.6e-10	185	243	1msz
☑	RRM_1	0.13	448	508	1fj7
☐	DinI	4.7	126	192	1ghh
☐	CbiK	7.2	223	432	1qgo

B

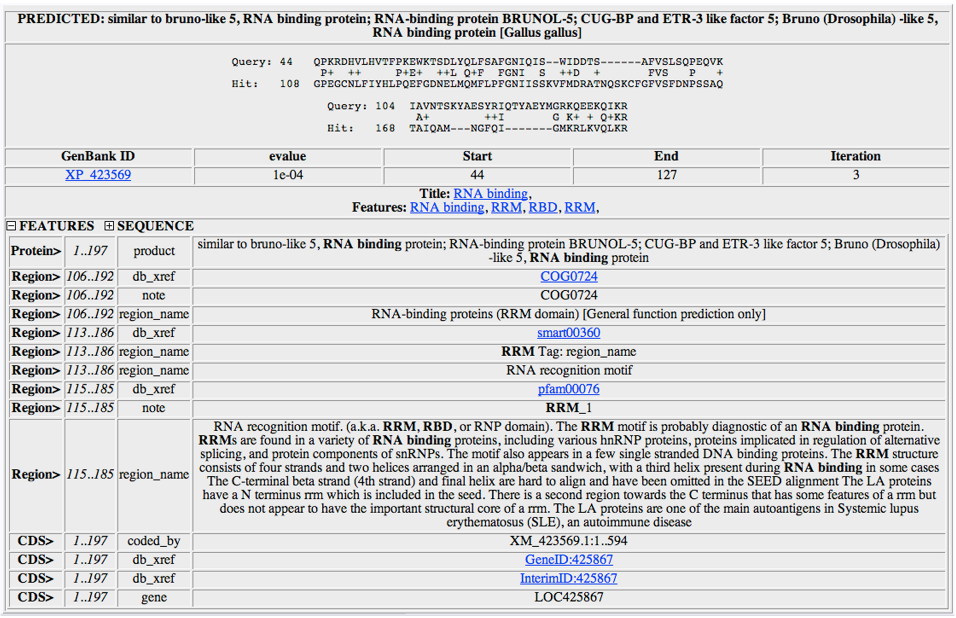

PREDICTED: similar to bruno-like 5, RNA binding protein; RNA-binding protein BRUNOL-5; CUG-BP and ETR-3 like factor 5; Bruno (Drosophila) -like 5, RNA binding protein [Gallus gallus]

```
Query: 44    QPKRDHVLHVTFPKEWKTSDLYQLFSAFGNIQIS--WIDDTS------AFVSLSQPEQVK
              P+  ++      P+E+  ++L Q+F  FGNI  S  ++D  +        FVS   P   +
Hit:   108   GPEGCNLFIYHLPQEFGDNELMQMFLPFGNIISSKVFMDRATNQSKCFGFVSFDNPSSAQ

Query: 104   IAVNTSKYAESYRIQTYAEYMGRKQEEKQIKR
               A+           ++I          G K+ + Q+KR
Hit:   168   TAIQAM---NGFQI-------GMKRLKVQLKR
```

GenBank ID	evalue	Start	End	Iteration
XP_423569	1e-04	44	127	3

Title: RNA binding,
Features: RNA binding, RRM, RBD, RRM,

⊟ FEATURES ⊞ SEQUENCE

**Protein>**	*1..197*	product	similar to bruno-like 5, **RNA binding** protein; RNA-binding protein BRUNOL-5; CUG-BP and ETR-3 like factor 5; Bruno (Drosophila) -like 5, **RNA binding** protein
**Region>**	*106..192*	db_xref	COG0724
**Region>**	*106..192*	note	COG0724
**Region>**	*106..192*	region_name	RNA-binding proteins (RRM domain) [General function prediction only]
**Region>**	*113..186*	db_xref	smart00360
**Region>**	*113..186*	region_name	**RRM** Tag: region_name
**Region>**	*113..186*	region_name	RNA recognition motif
**Region>**	*115..185*	db_xref	pfam00076
**Region>**	*115..185*	note	**RRM**_1
**Region>**	*115..185*	region_name	RNA recognition motif. (a.k.a. **RRM**, **RBD**, or RNP domain). The **RRM** motif is probably diagnostic of an **RNA binding** protein. **RRMs** are found in a variety of **RNA binding** proteins, including various hnRNP proteins, proteins implicated in regulation of alternative splicing, and protein components of snRNPs. The motif also appears in a few single stranded DNA binding proteins. The **RRM** structure consists of four strands and two helices arranged in an alpha/beta sandwich, with a third helix present during **RNA binding** in some cases The C-terminal beta strand (4th strand) and final helix are hard to align and have been omitted in the SEED alignment The LA proteins have a N terminus rrm which is included in the seed. There is a second region towards the C terminus that has some features of a rrm but does not appear to have the important structural core of a rrm. The LA proteins are one of the main autoantigens in Systemic lupus erythematosus (SLE), an autoimmune disease
**CDS>**	*1..197*	coded_by	XM_423569.1:1..594
**CDS>**	*1..197*	db_xref	GeneID:425867
**CDS>**	*1..197*	db_xref	InterimID:425867
**CDS>**	*1..197*	gene	LOC425867

C

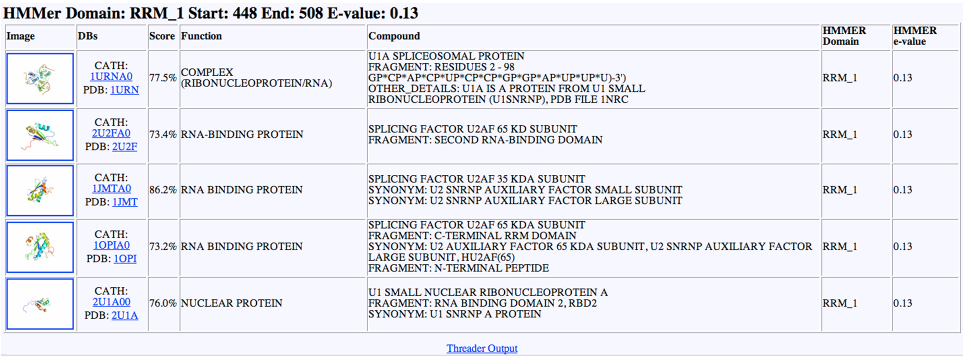

HMMer Domain: RRM_1 Start: 448 End: 508 E-value: 0.13

Image	DBs	Score	Function	Compound	HMMER Domain	HMMER e-value
	CATH: 1URNA0 PDB: 1URN	77.5%	COMPLEX (RIBONUCLEOPROTEIN/RNA)	U1A SPLICEOSOMAL PROTEIN<br>FRAGMENT: RESIDUES 2 - 98<br>GP*CP*AP*CP*UP*CP*CP*GP*GP*AP*UP*UP*U)-3')<br>OTHER_DETAILS: U1A IS A PROTEIN FROM U1 SMALL RIBONUCLEOPROTEIN (U1SNRNP), PDB FILE 1NRC	RRM_1	0.13
	CATH: 2U2FA0 PDB: 2U2F	73.4%	RNA-BINDING PROTEIN	SPLICING FACTOR U2AF 65 KD SUBUNIT<br>FRAGMENT: SECOND RNA-BINDING DOMAIN	RRM_1	0.13
	CATH: 1JMTA0 PDB: 1JMT	86.2%	RNA BINDING PROTEIN	SPLICING FACTOR U2AF 35 KDA SUBUNIT<br>SYNONYM: U2 SNRNP AUXILIARY FACTOR SMALL SUBUNIT<br>SYNONYM: U2 SNRNP AUXILIARY FACTOR LARGE SUBUNIT	RRM_1	0.13
	CATH: 1OPIA0 PDB: 1OPI	73.2%	RNA BINDING PROTEIN	SPLICING FACTOR U2AF 65 KDA SUBUNIT<br>FRAGMENT: C-TERMINAL RRM DOMAIN<br>SYNONYM: U2 AUXILIARY FACTOR 65 KDA SUBUNIT, U2 SNRNP AUXILIARY FACTOR LARGE SUBUNIT, HU2AF(65)<br>FRAGMENT: N-TERMINAL PEPTIDE	RRM_1	0.13
	CATH: 2U1A00 PDB: 2U1A	76.0%	NUCLEAR PROTEIN	U1 SMALL NUCLEAR RIBONUCLEOPROTEIN A<br>FRAGMENT: RNA BINDING DOMAIN 2, RBD2<br>SYNONYM: U1 SNRNP A PROTEIN	RRM_1	0.13

Threader Output
