# Supplementary material for: ProFAT: a web-based tool for the functional annotation of protein sequences
Source: BMC Bioinformatics. 2006 Oct 23;7:466. doi: 10.1186/1471-2105-7-466 (PMC1636073; doi:10.1186/1471-2105-7-466)
Supplement: Additional File 7 — ProFAT result for human protein LOC84060, which is predicted to have a weakly conserved RRM domain. [file 1471-2105-7-466-S7.pdf]

A

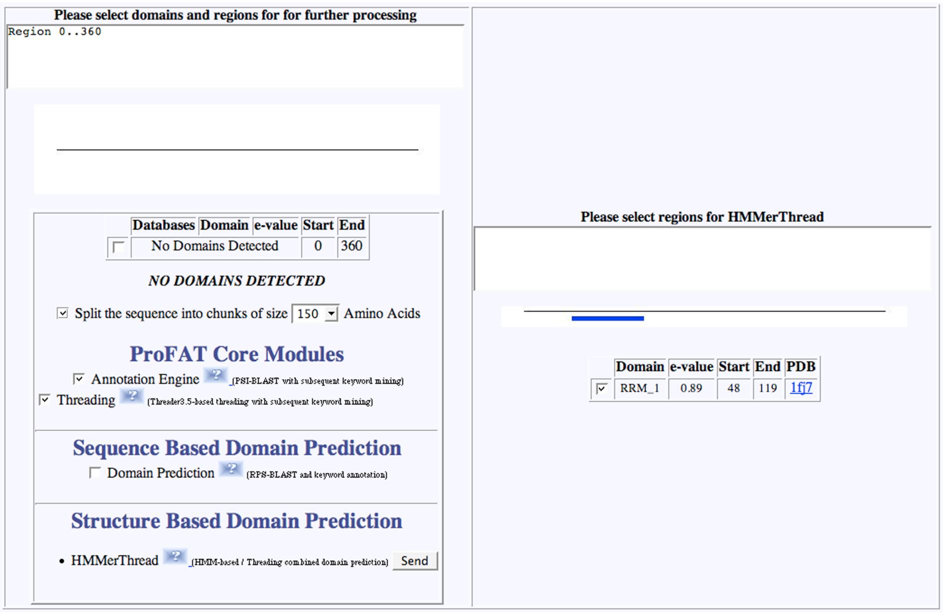

Please select domains and regions for for further processing
Region 0..360
Databases Domain e-value Start End
No Domains Detected 0 360
NO DOMAINS DETECTED
Split the sequence into chunks of size 150 Amino Acids
ProFAT Core Modules
Annotation Engine (PSI-BLAST with subsequent keyword mining)
Threading (Threader3.5-based threading with subsequent keyword mining)
Sequence Based Domain Prediction
Domain Prediction (RPS-BLAST and keyword annotation)
Structure Based Domain Prediction
HMMerThread (HMM-based / Threading combined domain prediction) Send
Please select regions for HMMerThread
Domain e-value Start End PDB
RRM_1 0.89 48 119 1fj7

B

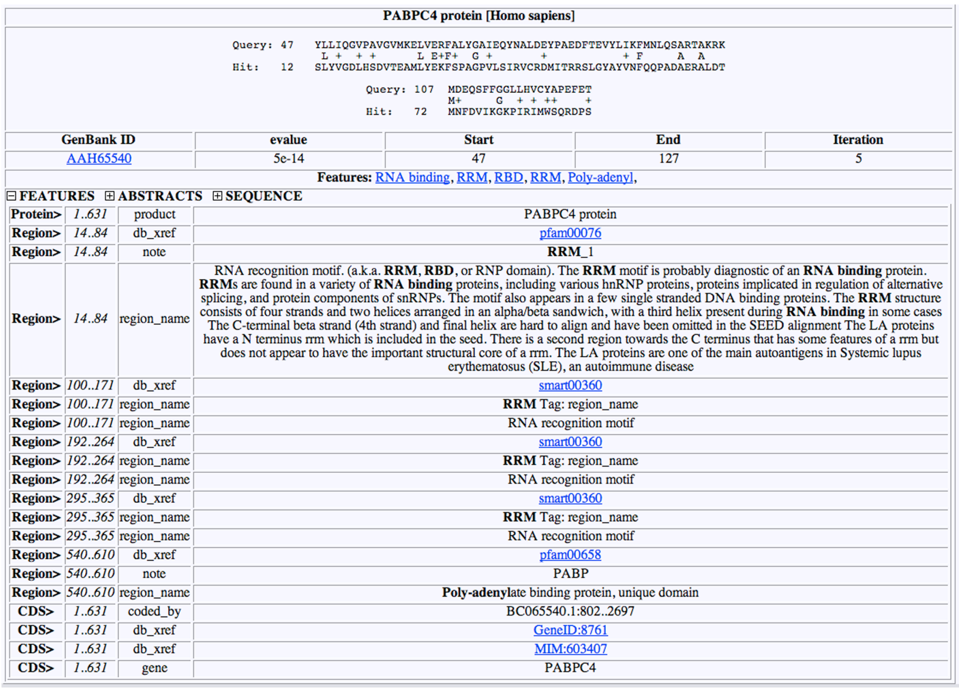

PABPC4 protein [Homo sapiens]
Query: 47 YLLIQGVPAVGVMKELVERFALYGAIEQYNALDEYPAEDFTEVYLIKFMNLQSARTAKRK
L + + + L E+F+ G + + + F A A
Hit: 12 SLYVGDLHSDVTEAMLYEKFSPAGPVLSIRVCRDMITRRSLGYAYVNFQQPADAERALDT
Query: 107 MDEQSFFGGLLHVCYAPEFET
M+ G + + ++ +
Hit: 72 MNFDVIKGKPIRIMWSQRDPS
GenBank ID evalue Start End Iteration
AAH65540 5e-14 47 127 5
Features: RNA binding, RRM, RBD, RRM, Poly-adenyl,
FEATURES ABSTRACTS SEQUENCE
Protein> 1..631 product PABPC4 protein
Region> 14..84 db_xref pfam00076
Region> 14..84 note RRM_1
Region> 14..84 region_name RNA recognition motif. (a.k.a. RRM, RBD, or RNP domain). The RRM motif is probably diagnostic of an RNA binding protein. RRMs are found in a variety of RNA binding proteins, including various hnRNP proteins, proteins implicated in regulation of alternative splicing, and protein components of snRNPs. The motif also appears in a few single stranded DNA binding proteins. The RRM structure consists of four strands and two helices arranged in an alpha/beta sandwich, with a third helix present during RNA binding in some cases The C-terminal beta strand (4th strand) and final helix are hard to align and have been omitted in the SEED alignment The LA proteins have a N terminus rrm which is included in the seed. There is a second region towards the C terminus that has some features of a rrm but does not appear to have the important structural core of a rrm. The LA proteins are one of the main autoantigens in Systemic lupus erythematosus (SLE), an autoimmune disease
Region> 100..171 db_xref smart00360
Region> 100..171 region_name RRM Tag: region_name
Region> 100..171 region_name RNA recognition motif
Region> 192..264 db_xref smart00360
Region> 192..264 region_name RRM Tag: region_name
Region> 192..264 region_name RNA recognition motif
Region> 295..365 db_xref smart00360
Region> 295..365 region_name RRM Tag: region_name
Region> 295..365 region_name RNA recognition motif
Region> 540..610 db_xref pfam00658
Region> 540..610 note PABP
Region> 540..610 region_name Poly-adenylate binding protein, unique domain
CDS> 1..631 coded_by BC065540.1:802..2697
CDS> 1..631 db_xref GeneID:8761
CDS> 1..631 db_xref MIM:603407
CDS> 1..631 gene PABPC4

C

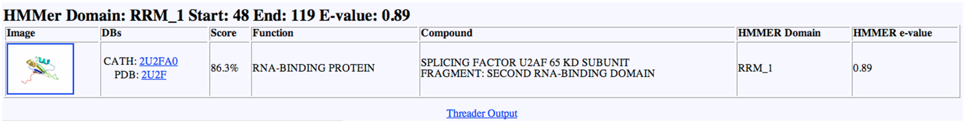

HMMer Domain: RRM_1 Start: 48 End: 119 E-value: 0.89
Image DBs Score Function Compound HMMER Domain HMMER e-value
CATH: 2U2FA0
PDB: 2U2F
86.3% RNA-BINDING PROTEIN SPLICING FACTOR U2AF 65 KD SUBUNIT FRAGMENT: SECOND RNA-BINDING DOMAIN RRM_1 0.89
Threader Output
