# Supplementary material for: ProFAT: a web-based tool for the functional annotation of protein sequences
Source: BMC Bioinformatics. 2006 Oct 23;7:466. doi: 10.1186/1471-2105-7-466 (PMC1636073; doi:10.1186/1471-2105-7-466)
Supplement: Additional File 8 — Original ProFAT results for the human protein LOC79969, which has a predicted acetyltransferase domain. [file 1471-2105-7-466-S8.pdf]

A

Please select regions for HMMerThread

Acetyltransf_1 - 1ghe 1i12 1i21 1i1d 1j4j 1ib1 1l0c 1m1d 1kuv

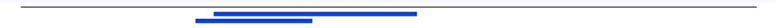

	Domain	e-value	Start	End	PDB
☑	Acetyltransf_1	2.6	85	174	1ghe
☐	PurS	5.5	77	128	1gtd

Submit

B

HMMer Domain: Acetyltransf_1 Start: 85 End: 174 E-value: 2.6

Image	DBs	Score	Function	Compound	HMMER Domain	HMMER e-value
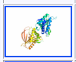	CATH: 1GHEA0 PDB: 1GHE	82.3%	TRANSFERASE	ACETYLTRANSFERASE SYNONYM: TABTOXIN RESISTANCE PROTEIN	Acetyltransf_1	2.6
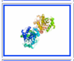	CATH: 1QSMA0 PDB: 1QSM	87.2%	TRANSFERASE	HPA2 HISTONE ACETYLTRANSFERASE	Acetyltransf_1	2.6
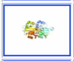	CATH: 1VHSA0 PDB: 1VHS	79.7%	STRUCTURAL GENOMICS, UNKNOWN FUNCTION	SIMILAR TO PHOSPHINOTHRICIN ACETYLTRANSFERASE	Acetyltransf_1	2.6
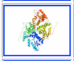	CATH: 1ON0A0 PDB: 1ON0	77.8%	STRUCTURAL GENOMICS, UNKNOWN FUNCTION	YYCN PROTEIN	Acetyltransf_1	2.6
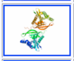	CATH: 1TIQA0 PDB: 1TIQ	86.6%	STRUCTURAL GENOMICS, TRANSCRIPTION	PROTEASE SYNTHASE AND SPORULATION NEGATIVE REGULATORY PROTEIN PAI 1	Acetyltransf_1	2.6
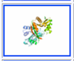	CATH: 1S3ZA0 PDB: 1S3Z	83.6%	TRANSFERASE	AMINOGLYCOSIDE 6'-N-ACETYLTRANSFERASE SYNONYM: AMINOGLYCOSIDE N-ACETYLTRANSFERASE AAC(6')-IY	Acetyltransf_1	2.6
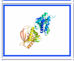	CATH: 1GHEB0 PDB: 1GHE	81.5%	TRANSFERASE	ACETYLTRANSFERASE SYNONYM: TABTOXIN RESISTANCE PROTEIN	Acetyltransf_1	2.6
