# Supplementary material for: ProFAT: a web-based tool for the functional annotation of protein sequences
Source: BMC Bioinformatics. 2006 Oct 23;7:466. doi: 10.1186/1471-2105-7-466 (PMC1636073; doi:10.1186/1471-2105-7-466)
Supplement: Additional File 9 — Figure legends for Additional files 3 to 8. [file 1471-2105-7-466-S9.pdf]

Additional file 9: Figure legends for Additional files 3 - 8

Additional file 3

(A) ProFAT preprocessing results for human Hook3. RPS-BLAST identified an SbcC domain within the coiled-coil region of Hook3 (left side). The N-terminal 1-153 amino acids were selected for further processing using the *Annotation* and *Threading Engine*. HMMer identified among other domains a CH domain between amino acids 12 to 120 in Hook3 (right side), which was selected for threading. **(B)** ProFAT *Annotation Engine* results for human Hook3. Hook3 N-terminal region is related to Arabidopsis Fimbrin. The *Annotation Engine* identified Fimbrin as a potentially similar, biologically relevant hit. **(C)** ProFAT *HMMerThread* results for the predicted CH domain of human Hook3. Threader identified two CH domain structures as potential homologues of Hook3 with a confidence level of nearly 70%.

Additional file 4

(A) ProFAT preprocessing results for human Kpl2. The domain search identified a N-terminal domain of unknown function, DUF1042, an adjacent coiled-coil domain, as well as an adenylate kinase domain (left side). DUF1042 was selected for further processing. HMMer identified a CH domain within the first 150 amino acids of the protein that was sent to threading (right side). (**B**) ProFAT *Annotation Engine* results for human Kpl2. The *Annotation Engine* identified among others the CH domain of Mal3 and Arabidopsis EB1. (**C**) ProFAT *HMMerThread* results for human Kpl2. *HMMerThread* identified the structures of the CH domains of T-Fimbrin as structurally related to the predicted CH domain of Kpl2 with a confidence of around 80%.

Additional file 5

(A) ProFAT preprocessing results for human EPS8L3. The domain search of ProFAT detected an EPS/PTB domain in the N-terminus along with a SH3 domain in human EPS8L3 (left side). The C-terminal region adjacent to the SH3 domain was selected for further processing. HMMer detected a SAM_1 domain in the C-terminus of the protein, which was sent to Threader (right side). **(B)** ProFAT *Annotation Engine* results for human EPS8L3. Among other hits, ProFAT's *Annotation Engine* identified the SAM domain of a predicted protein from chicken, as well as the SAM domain of Kinase Suppressor of Ras from Rat that contained keywords from the provided list. **(C)** ProFAT *HMMerThread* results for human EPS8L3. For the predicted SAM_1 domain of EPS8L3, Threader detected the structure of a SAM domain from EPHB2 with over 90% certainty.

Additional file 6

(A) ProFAT preprocessing results for human PARN. RPS-BLAST detected a CAF1 domain in the N-terminus of the protein, which is split by a PARN_RH3 domain (left side). The C-terminal region of the protein was selected for further processing. HMMer detected among other domains a RRM_1 domain, which was submitted to Threader (right side). **(B)** ProFAT *Annotation Engine* results for human PARN. The *Annotation Engine* found among others the Bruno-like 5 RNA binding protein from chicken as a relative of PARN. The detected similarity lies within the RRM domain of Bruno-like 5. **(C)** ProFAT *HMMerThread* results for human PARN. *HMMerThread* identified several RRM motifs containing domains from crystallized proteins with up to 86% confidence.

Additional file 7

(A) ProFAT preprocessing results for human LOC84060. RPS-BLAST found no conserved domains in this protein (left side). The entire sequence was submitted for further processing. HMMer detected a RRM_1 domain in the N-terminal part of the protein, which was sent to Threader (right side). **(B)** ProFAT *Annotation Engine* results for LOC84606. Among other relatives, ProFAT's *Annotation Engine* identified the RRM domain of RNA-binding protein PABPC4 as related in sequence. **(C)** ProFAT *HMMerThread* results for human LOC84060. *HMMerThread* found similarity between LOC840606 and the second RRM domain of splicing factor U2AF with over 85% certainty.

Additional file 8

(A) ProFAT preprocessing results for human LOC79969. HMMer identified an Acetyltransferase_1 domain in the N-terminal half of the protein, which was sent to Threader for further processing. **(B)** ProFAT *HMMerThread* results for human LOC79969. *HMMerThread* detected similarity between LOC79969 and several acetyltransferase domains with high confidence.
