# Supplementary material for: ProFAT: a web-based tool for the functional annotation of protein sequences
Source: BMC Bioinformatics. 2006 Oct 23;7:466. doi: 10.1186/1471-2105-7-466 (PMC1636073; doi:10.1186/1471-2105-7-466)
Supplement: Additional File 10 — Table containing all accession numbers used for multiple sequence alignments in Figure 7. A detailed manual of the usage of the ProFAT web server containing screenshots of typical ProFAT results is available online [35]. [file 1471-2105-7-466-S10.pdf]

Additional file 10: Accession Numbers

Figure 7 A: CH-domain alignment

**ScFimbrin**	[GenBank:P32599]
**RnPlastin3**	[GenBank:Q63598]
**HsFilaminA**	[GenBank:P21333]
**HsIQGAP1**	[GenBank:P46940]
**DdFimbrin**	[GenBank:P54680]
**DmCG8953**	[GenBank:AAF55366]
**CeC10G11.7**	[GenBank:AAB42247]
**Hook1**	[GenBank:NP_056972]
**Hook2**	[GenBank:NP_037444]
**Hook3**	[GenBank:NP_115786]
**HsKPL2**	[GenBank:NP_653323]
**SpuKPL2**	[GenBank:XP_781844]
**CrCPC1**	[GenBank:AAT40991]

Figure 7 B: SAM-domain alignment

**RnEPHA3**	[GenBank:O08680]
**CeF13B10.1a**	[GenBank:CAA90182]
**HsDLC-1**	[GenBank:AAB87700]
**CeF53F8.5**	[GenBank:CAB04458]
**HsLiprina2**	[GenBank:AAC26100]
**MmTA*p63a**	[GenBank:AAC62641]
**SpMKH1**	[GenBank:Q10407]
**HsSLP76**	[GenBank:Q13094]
**CeC11E4.6**	[GenBank:CAB02658]
**HsEPS8L3**	[GenBank:NP_620641]
**MmEPS8L3**	[GenBank:AAH14734]
**TnEPS8L3**	[GenBank:CAG06824]
**HsEPS8L2**	[GenBank:AAI01482]
**MmEPS8L2**	[GenBank:NP_573454]
**HsEPS8**	[GenBank:NP_004438]
**MmEPS8**	[GenBank:NP_031971]
**GgEPS8**	[GenBank:XP_416405]
**DrEPS8**	[GenBank:AAH47814]
**DmEPS8**	[GenBank:NP_650585]
**CeEPS8**	[GenBank:NP_502802]

Figure 7 C: RRM-domain alignment

**HsTia1**	[GenBank:P31483]
**AtRT19**	[GenBank:P39697]
**NsRoc5**	[GenBank:P19684]
**DmSrp55**	[GenBank:P26686]
**DmElav**	[GenBank:P16914]
**HsParn**	[GenBank:NP_002573]
**AmParn**	[GenBank:XP_392167]
**AtParn**	[GenBank:Q9LG26]
**HsLOC84060**	[GenBank:NP_115496]
**DrLOC402897**	[GenBank:NP_991167]
**DmHDC05885**	[GenBank:DAA02438.1]

Figure 7 D: Acetyl-transferase alignment

**MtCAB08501**	[GenBank:CAB08501]
**TaCAA12252**	[GenBank:CAA12252]
**AbIAAT**	[GenBank:P26945]
**MtRimI**	[GenBank:NP_217937]
**MjMJ1207**	[GenBank:Q58604]
**EcAAA62661**	[GenBank:AAA62661]
**MthAAB85181**	[GenBank:AAB85181]
**HsFus2**	[GenBank:Q93015]
**HsLOC79969**	[GenBank:NP_079185]
**DmCG3967**	[GenBank:NP_648309]
**CeMec-17**	[GenBank:NP_501337]
